# ROS-independent ER stress-mediated NRF2 activation promotes warburg effect to maintain stemness-associated properties of cancer-initiating cells

**DOI:** 10.1038/s41419-017-0250-x

**Published:** 2018-02-07

**Authors:** Ching-Wen Chang, Yu-Syuan Chen, Yeou-Guang Tsay, Chia-Li Han, Yu-Ju Chen, Cheng-Chieh Yang, Kai-Feng Hung, Chao-Hsiung Lin, Tsung-Yen Huang, Shou-Yen Kao, Te-Chang Lee, Jeng-Fan Lo

**Affiliations:** 1 Institute of Oral Biology, National Yang-Ming University, Taipei, Taiwan; 2 Department of Biotechnology and Laboratory Science in Medicine, National Yang-Ming University, Taipei, Taiwan; 3 Institute of Biochemistry and Molecular Biology, National Yang-Ming University, Taipei, Taiwan; 4 Institute of Chemistry, Academia Sinica, Taipei, Taiwan; 5 Department of Dentistry, School of Dentistry, National Yang-Ming University, Taipei, Taiwan; 6 Department of Medical Research and Education, Taipei Veterans General Hospital, Taipei, Taiwan; 7 Department of Life Sciences and Institute of Genome Sciences, National Yang-Ming University, Taipei, Taiwan; 8 Institute of Biomedical Sciences, Academia Sinica, Taipei, Taiwan; 9 Genome Research Center, National Yang-Ming University, Taipei, Taiwan; 10 Graduate Institute of Chinese Medical Science and Institute of Medical Science, China Medical University, Taichung, Taiwan; 11 Department of Dentistry, Taipei Veterans General Hospital, Taipei, Taiwan; 12 National Yang-Ming University VGH Genome Research Center, Taipei, Taiwan

## Abstract

Cancer-initiating cells (CICs) are responsible for tumor initiation, progression, and therapeutic resistance; moreover, redox homeostasis is important in regulating cancer stemness. Previously, we have identified that cancer cells containing low intracellular reactive oxygen species levels (ROS^Low^ cells) display enhanced features of CICs. However, the specific metabolic signatures of CICs remain unclear and are required for further characterization by systemic screenings. Herein, we first showed CICs mainly relying on glycolysis that was important for the maintenance of stemness properties. Next, we revealed that NRF2, a master regulator of antioxidants, was able to maintain low intracellular ROS levels of CICs, even though in the absence of oxidative stress. We further characterized that NRF2 activation was required for the maintenance of CICs properties. Of ROS^Low^ cells, NRF2 activation not only directly activates the transcription of genes encoding glycolytic enzymes but also inhibited the conversion of pyruvate to acetyl-CoA by directly activating pyruvate dehydrogenase kinase 1 (PDK1) to lead to inhibition of tricarboxylic acid (TCA) cycle; therefore, to promote Warburg effect. A positive regulatory ROS-independent ER stress pathway (GRP78/p-PERK/NRF2 signaling) was identified to mediate the metabolic shift (Warburg effect) and stemness of CICs. Lastly, co-expression of p-PERK and p-NRF2 was significantly associated with the clinical outcome. Our data show that NRF2 acting as a central node in the maintenance of low ROS levels and stemness associated properties of the CICs, which is significantly associated with the clinical outcome, but independent from ROS stress. Future treatments by inhibiting NRF2 activation may exhibit great potential in targeting CICs.

## Introduction

Cancer-initiating cells (CICs) exploit the characteristics of self-renewal and differentiation to drive tumor growth and progression^1^. Previously, we have enriched and identified head and neck CICs (HN-CICs) through sphere culture^2^. Our most recent study shows that a subset of HN-CICs contains lower ROS levels. Consequently, the sorted ROS^Low^ cells possess enhanced stemness properties and tumorigenicity and acquire a quiescent state. Furthermore, compared with ROS^Low^ cells, the other subset of HN-CICs with high ROS levels (the ROS^High^ cells) are more proliferative but exhibit the less self-renewal capacity^3^. Given the importance of redox homeostasis in regulating the stemness of CICs, we need to understand the unique physiology to balance the ROS levels and stemness of CICs.

In various cancers, CICs are considered highly heterogeneous and harbor a distinct metabolic phenotype in terms of stemness features^4^. Of note, ROS is intimately tied to cellular metabolic phenotype^5^. Additionally, mitochondria are the major source of ROS production through oxidative phosphorylation (OXPHOS)^5^. Interestingly, CICs have been described as preferentially relying on the Warburg effect or OXPHOS in a cancer type-dependent manner^6–9^. Warburg effect not only provides sufficient energy demands but also minimizes ROS production in mitochondria^8, 10^. Furthermore, we recently have demonstrated that ROS^Low^ cells highly express the high-affinity glucose transporter, GLUT3^3^. Indeed, metabolic reprogramming of cancer cells tightly regulates defense against oxidative stress, thus promoting tumorigenesis and chemoresistance^11^.

From an initial screen of molecular mechanisms known to play a role in mediating CICs metabolism, we found a transcription factor NRF2 activity correlated with the Warburg effect (see the following contexts). NRF2 is a master regulator of ROS-scavenging enzymes^12^. Indeed, NRF2 has been considered to regulate the self-renewal of various kinds of normal stem cells. A recent study demonstrated that NRF2 is required for the switch to glycolysis by promoting HIFα activation in iPSC reprogramming^13^. Further, NRF2 has shown prognostic significance in many solid tumors^14, 15^. Nevertheless, the mechanisms by which NRF2 controls cell metabolism that maintain redox homeostasis, and therefore sustains CICs properties, remain to be elucidated. Furthermore, the molecular mechanisms by which NRF2 can be activated in CICs also remain elusive.

Our current study provides several insights into distinct subsets of cancer cells with different ROS levels, in which metabolic reprogramming and activation of NRF2 signaling are the main mechanisms regulating cancer stemness.

## Results

### Reprogrammed glucose metabolism in HN-CICs

**Fig. 1:**
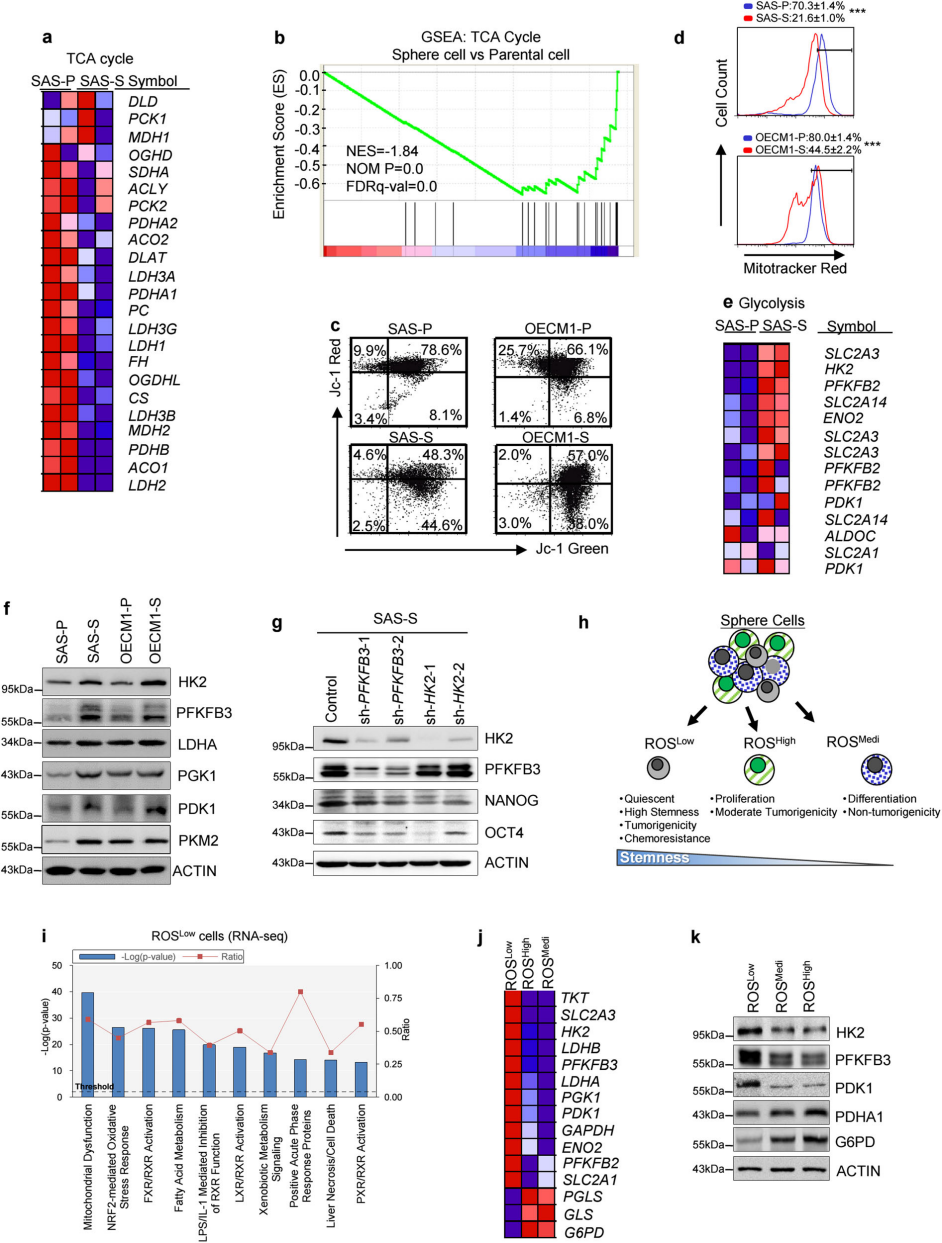
Head and neck CICs relying on the Warburg effect. **a** Heatmap depicting expression of 24 genes encoding tricarboxylic acid (TCA) cycle pathway in parental (SAS-P) and sphere cells (SAS-S). **b** Gene Set Enrichment Analysis (GSEA) of a 24-gene set comprising (TCA) cycle pathway in SAS-P versus SAS-S (*NES* normalized enrichment score; *P* = nominal *P*-value; *FDR* false discovery rate). **c** The mitochondrial membrane potentials (Δψ, red/green ratios) of the parental and sphere cells were determined using JC-1 staining followed by FACS. (OECM1 parental (OECM1-P) and OECM1 sphere cells (OECM1-S)). **d** The mitochondrial masses of the parental and sphere cells were determined using MitoTracker Red staining followed by FACS. **e** Heatmap depicting expression of genes encoding glycolytic pathway in parental and sphere cells. **f** Immunoblots detecting the protein level of glycolytic enzymes in parental and sphere cells. **g** Immunoblots showing the expression level of HK2, PFKFB3, NANOG and OCT4 proteins in control, sh-*HK2* or sh-*PFKFB3* sphere cells, respectively. **h** Schematic depicting features of ROS^Low^, ROS^High^, and ROS^Medi^ cells. **i** Top 10 toxicology pathway lists identified in ROS^Low^ cells from RNA-Seq analysis. *P*-value, Fisher’s exact test. Threshold, the minimum significance level (−Log [*P*-value] as 1.25). Ratio, the molecules from the data set divided by the total molecules that map to the toxicology pathway from within the IPA software. **j** Heatmap depicting expression of genes encoding glycolytic pathway in ROS^Low^, ROS^Medi^, and ROS^High^ cells (*P* < 0.001, Fisher’s exact test). **k** Immunoblots detecting the glycolytic enzymes and epithelial differentiation markers in sorted ROS^Low^, ROS^Medi^, and ROS^High^ cells

Previously, we and others demonstrated that CICs, enriched within the sphere cells under serum-free culture conditions of cancer cells^2, 16^. To unravel the metabolic features of CICs, we first investigated possible pathways of glucose metabolism in HN-CICs. Initially, the expression profile of TCA cycle-related genes in sphere cells (SAS-S) and in parental cells (SAS-P) was analyzed by gene set enrichment analyses. Notably, TCA cycle-related genes were significantly downregulated in sphere cells (Figs. 1a, b). We further confirmed these results by measuring the mitochondrial membrane potentials of the parental and sphere cells with JC-1 staining. Red JC-1 aggregates are typical of healthy mitochondria^17^. Indeed, the sphere cells had fewer red JC-1 aggregates than the parental cells that indicate the occurrence of mitochondrial depolarization within the sphere cells (Fig. 1c). In addition, we found an approximately 2–3 folds reduction in the mitochondrial mass in sphere cells versus parental cells (Fig. 1d; SAS-P: 70.3% vs. SAS-S: 21.6%; OECM1-P: 80% vs. OECM1-S: 44.5%). Strikingly, the sphere cells displayed a higher expression of glycolytic enzymes in order to promote glycolysis (Figs. 1e, f and Figure S1a,S1b). Given that radiation-resistant cells have been reported to have characteristics of cancer stemness^18^, we set out to evaluate the correlation between glycolytic enzymes expression profile and radioresistance properties. Interestingly, expression of glycolytic enzymes was significantly higher in radiation-resistant cells than in parental cells (Figure S1a). Next, we wanted to address whether genetically or pharmacologically inhibition of glycolysis would abrogate the stemness properties of CICs. Genetic inhibition of HK2 or PFKFB3 by shRNAi, respectively, both reduced the expression of stemness markers (NANOG and OCT4) in SAS sphere cells (Fig. 1g). Furthermore, pharmacologic inhibition of glycolysis by addition of 2-deoxyglucose (2-DG) showed the most significant effect on increasing cytotoxic effects and decreasing expression of cancer stemness marker (OCT4 and CD44) in sphere cells (Figure S1c-S1e).

We recently demonstrate that ROS^Low^ cells were quiescent but exhibited the highest tumorigenicity; whereas ROS^High^ cells showed higher proliferative activity and moderate tumorigenicity (Fig. 1h)^3^. To gain the mechanistic insight into distinct subpopulation cells, we performed RNA-Seq analyses with sorted ROS^Low^ cells versus ROS^High^ cells to examine associated changes in cellular pathways and functions. The transcriptome of the ROS^Low^ cells displayed a very distinct expression profile compared with that of the ROS^High^ and ROS^Medi^ subpopulation cells (Figure S1f). Interestingly, the metabolic process was highlighted as having the first ranking among the significant differences (Figure S1g). Specifically, we also discovered the major mechanistic features, mitochondrial dysfunction, was up-regulated in the ROS^Low^ cells (Fig. 1i and Figure S1h). Consistent with these results, the ROS^Low^ cells exhibited reduced mitochondrial membrane potentials than the ROS^High^ and ROS^Medi^ cells (Figure S1i). In addition, the glycolysis-related genes were significantly up-regulated in the ROS^Low^ cells (Fig. 1j,k). Finally, to further examine whether pharmacologic modulation of glycolysis contributed to cell death in ROS^Low^ cells, ROS^Low^ cells were treated with 2-DG. Treatment of 2-DG caused a significant increase in cell death in ROS^Low^ cells (Figure S1j).Collectively, these results suggest that HN-CICs may rely on the glycolytic pathway and exhibit impaired OXPHOS, which is consistent with a “Warburg effect” profile.

### Elevated NRF2 expression in HN-CICs

**Fig. 2:**
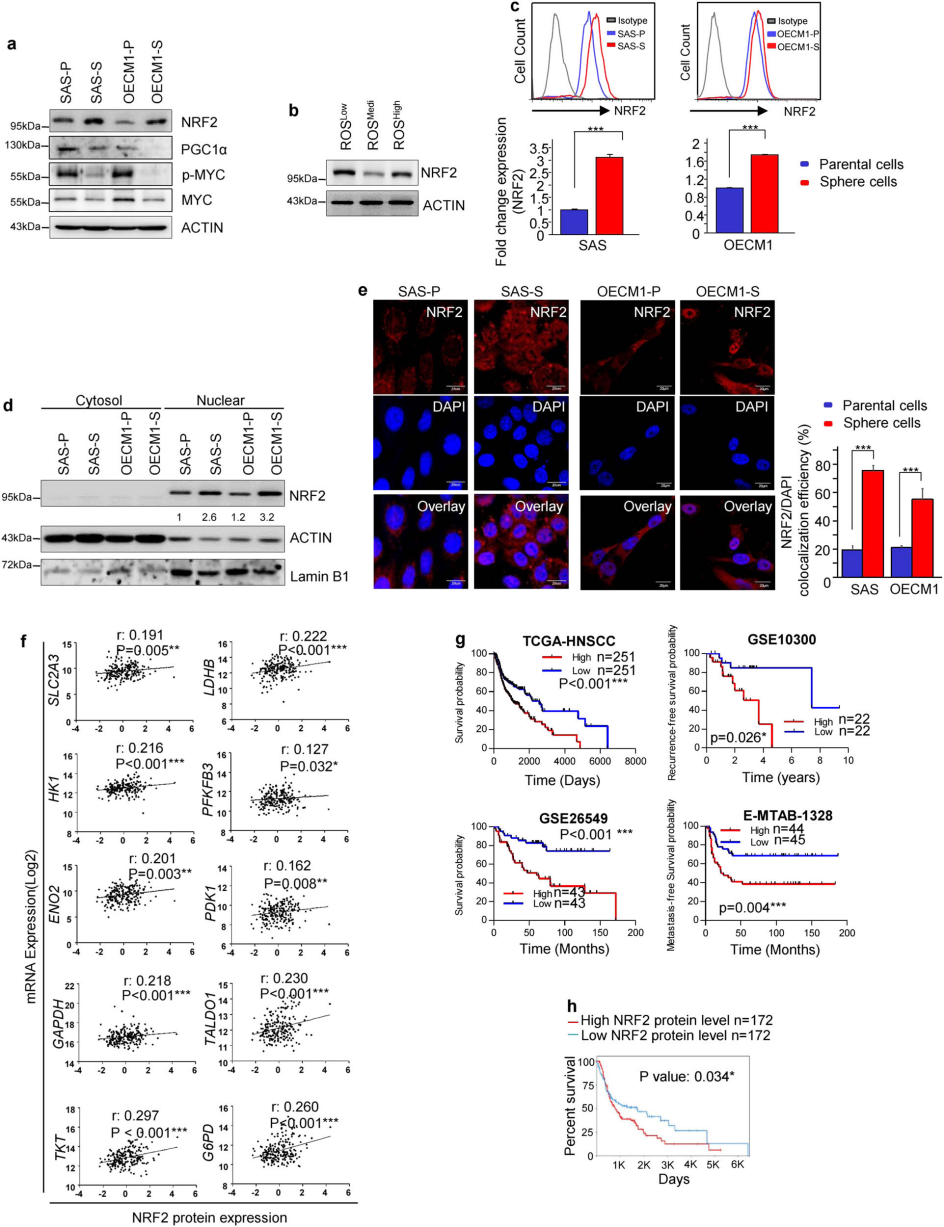
NRF2 is highly expressed in sphere cells compared to parental cells and critical for glycolytic metabolic signatures of HNSCC. **a** Immunoblots showing NRF2, PGC1α, and p-MYC protein levels in parental and sphere cells. **b** Immunoblots showing NRF2 protein level in ROS^Low^, ROS^Medi^, and ROS^High^ cells. **c** FACS analyses showing the profiles of p-NRF2-positive parental and sphere cells (*n* = 3 independent experiments; *t*-test, *** indicates *P* < 0.001). **d** NRF2, actin and lamin B1 protein levels in the nuclear and cytosolic fractions prepared from parental or sphere cells. **e** Immunofluorescence staining showing the subcellular localization of the NRF2 protein (red) and nuclei stain DAPI (blue) in parental and sphere cells. Quantification of the colocalization efficiency between NRF2 and DAPI confirming that NRF2 was present in sphere cell nucleus. **f** Correlation analyses between the protein level of NRF2 and mRNA level of glycolytic genes in HNSCC, according to the TCGA database, respectively. Correlation coefficients (*r*) and *P*-values are displayed (*n* = 346). Data were obtained from the cBioPortal website. **g** Kaplan–Meier analysis showing the overall survival of patients with Head and neck cancers according to the expression of seven glycolytic transcripts. Data were obtained from the SurvExpress website. **h** Head and neck cancers (*n* = 346) data obtained from TCGA database (The Cancer Proteome Atlas (TCPA) website) were used for Kaplan-Meier analysis to predict the overall survival of patients according to the expression of the NRF2 protein

To further elucidate the underlying molecular mechanisms to mediate metabolic regulation of redox homeostasis, we performed a screening of the candidate transcription factors (NRF2, PGC1α, and p-MYC), which are known to correlate with the metabolic phenotypes and the stress/antioxidant response in cancer cells^19–22^. Interestingly, we found that only the protein level of NRF2 but not others were up-regulated in sphere cells compared with that of parental cells (Fig. 2a). These data were consistent with the IPA analyses identifying that the activation of NRF2 signaling pathway was the most significantly affected pathways in ROS^Low^ cells (Fig. 1i). NRF2 activation in cells with different levels of ROS was further examined to validate the observation that low ROS levels were positively regulated by NRF2. It was shown that increased expression of the NRF2 protein in ROS^Low^ was observed in comparison with that in ROS^Medi^ or ROS^High^ cells (Fig. 2b). Moreover, the expression profile of NRF2-mediated downstream targets was also increased in SAS sphere cells compared with those in SAS parental cells (Figures S2a and S2b). Phosphorylation of NRF2 (p-NRF2) is required for nuclear translocation and activation of NRF2 to increase drug resistance^23^. Consistently, sphere cells contained more p-NRF2 expression than the parental cells did (Fig. 2c). Subsequently, immunoblotting and immunofluorescence revealed an approximately 3 folds increase in the amount of nuclear NRF2 in the sphere cells (Figs. 2d, e; immunofluorescence: SAS-P:18.6% vs. SAS-S:73.6%; OECM1-P: 21.2% vs. OECM1-S:56.0%). On the basis of these results, we focused on further characterizing the effect of NRF2 activation on glucose metabolism of CICs. It has been demonstrated that NRF2 directly activates PPP and glutamine metabolism pathway in cancer cells^24^. However, a relationship between NRF2 and glycolysis in CICs remains elusive. We first investigated the relationship between the expression of NRF2 and glycolytic genes in clinical patients by analyzing the TCGA database. A positive correlation was observed NRF2 protein expression with the expression of glycolytic or PPP genes (Fig. 2f). Further, higher glycolytic genes expression predicted poor survival for HNSCC cancer (Fig. 2g). In addition, the increased protein level of NRF2 was positively correlated with worse survival in cancer patients (Fig. 2h). In summary, NRF2 was highly expressed in both sphere cells and ROS^Low^ cells, which may be correlated with NRF2-mediated metabolic phenotypes. Therefore, we hypothesize that NRF2 activation is crucial for regulating the CIC metabolism.

### NRF2 directly activates glycolytic pathway and PPP in sphere cells

**Fig. 3:**
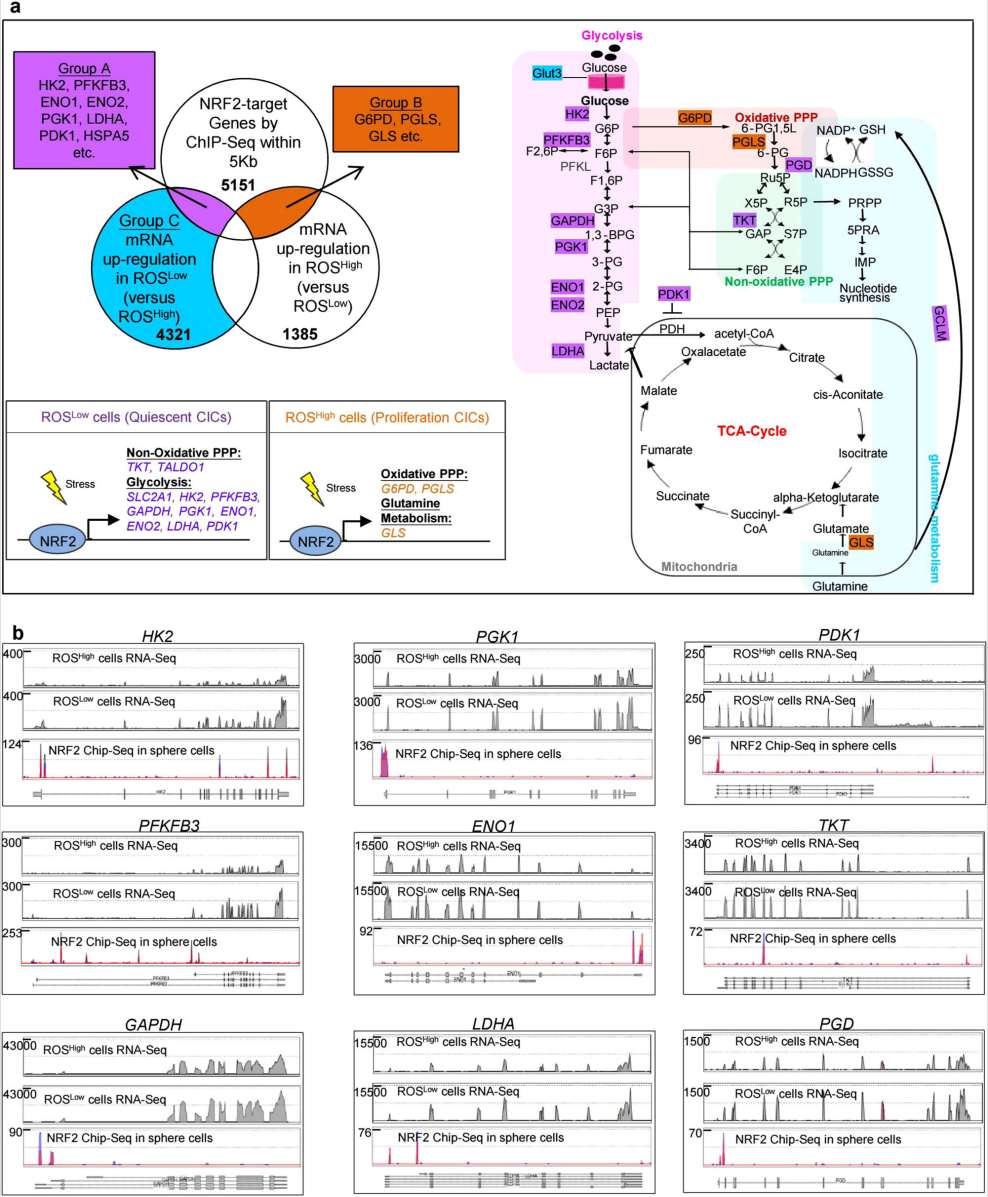
NRF2 directly activates glycolytic genes and reduces pyruvate entry into the TCA cycle to regulate the intracellular reduction/oxidation (redox) state. **a** (Upper left panel) Venn diagram depicting the overlap genes (Group A; purple) between the NRF2-bound genes (ChIP-Seq analyses) and genes with significantly altered expression in ROS^Low^ cells (blue). Otherwise, the Venn diagram depicts the overlap genes (Group B; orange) between NRF2-bound genes and genes with significantly altered expression in ROS^High^ cells. (Right panel) NRF2 mediated metabolic genes, involved in glycolysis and the non-oxidative PPP pathway in ROS^Low^ cells (Group A), or involved in the oxidative PPP and glutamine pathway in ROS^High^ cells (Group B). (Lower left panel) Summary of NRF2 regulating distinct metabolic pathways in ROS^Low^ cells (quiescent CICs) and ROS^High^ cells (proliferating CICs). **b** ChIP-Seq tracks of NRF2 in SAS sphere cells, assessing the binding of glycolysis and PPP. Location of ChIP-Seq peaks that were identified by using Strand NGS software. RNA-Seq traces (UCSC genome browser) for each gene in ROS^Low^ cells and ROS^High^ cells

For a better understanding of the role of NRF2 activation in regulating CICs metabolic phenotypes, global mapping of NRF2 binding sites on genomic loci of the sphere and parental cells using ChIP-Seq analyses was performed. We identified 1258 NRF2 genomic binding sites in parental SAS cells and 5151 NRF2 genomic binding sites in SAS sphere cells within 5 kb of the gene (Figures S3a and S3b). Of note, sphere cells showed a constitutive increase nuclear accumulation of NRF2 (Figs. 2d, e), which may, at least in part, explained the larger number of NRF2 binding sites identified in sphere cells rather than in the parental cells. Strikingly, NRF2-targeted sites in sphere cells (Ranking 10th) occupied substantially more ontology categories in the metabolic process than in parental cells (Ranking 16th) (Figure S3c). By combining the NRF2 ChIP-Seq and the ROS^Low^ and ROS^High^ cells-upregulated transcriptome datasets, we identified the genes that were directly regulated by NRF2 in the ROS^Low^ and ROS^High^ cells (Fig. 3a). Further, through integrating the NRF2 ChIP-Seq data from the sphere cells with the RNA-Seq data from the ROS^Low^ cells, we observed that NRF2 was able to directly activate the key enzymes in the glycolysis (*HK2*, and *PFKFB3* et al.), the checkpoint in regulating OXPHOS (*PDK1*), an enzyme in the non-oxidative PPP pathway (*TKT*) in sphere cells and these genes indeed were upregulated in ROS^Low^ cells (Fig. 3b). However, that bounding between NRF2 and genomic loci was lost in parental cells by ChIP-Seq analyses (Figure S3d). In contrast, the combined analysis of the NRF2 ChIP-Seq data from sphere cells and the RNA-Seq data from ROS^High^ cells revealed enrichments in oxidative PPP and glutamine metabolic targets (G6PD and GLS) (Figure S3e).

In summary, NRF2 activation not only directly activates the genes encoding glycolytic enzymes but also inhibits the TCA cycle by directly activating PDK1. These findings have led to the hypothesis that NRF2 may maintain low ROS levels within CICs by enhancing the Warburg effect.

### NRF2 activation promotes the “Warburg effect”

**Fig. 4:**
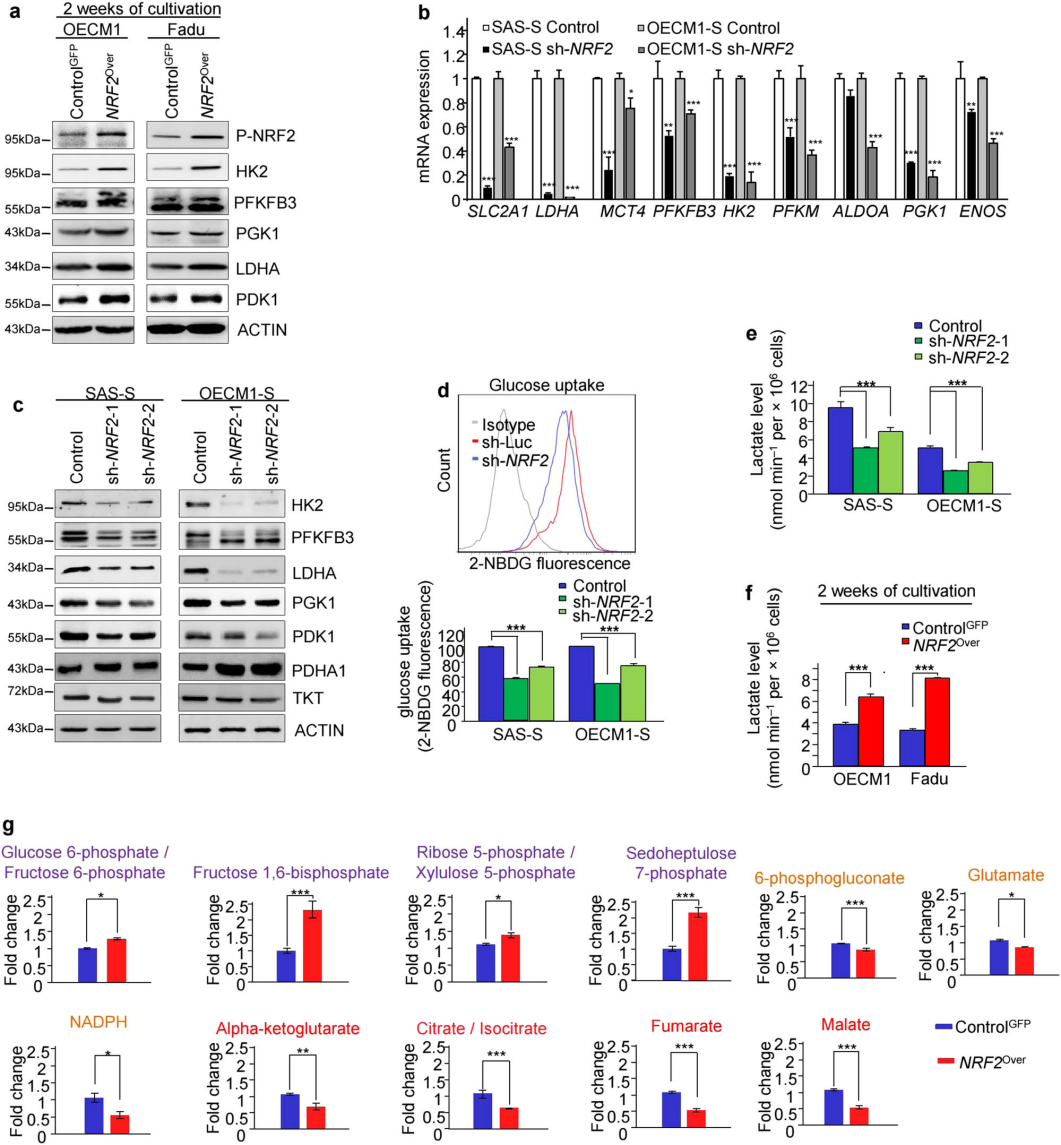
NRF2 upregulates Warburg effect in CICs. **a** Glycolytic enzyme protein levels in Control^GFP^ or *NRF2*^Over^ cells cultivated in selection medium for 2 weeks. **b** Total transcripts were prepared from sh-*NRF2* and Control sh-Luc sphere cells (SAS and OECM1 cells) and further analyzed by Real-time RT-PCR. **c** Total proteins, prepared from sh*-NRF2* and control sphere cells (SAS-S, OECM1-S), were analyzed by immunoblotting with the indicated antibodies. **d** Glucose uptake levels in sh*-NRF2* and Control sphere cells were measured by detecting the uptake of fluorescent 2-NBDG dye. **e** Intracellular lactate levels measured in sh*-NRF2* and control sphere cells. **f** Intracellular lactate levels measured in Control^GFP^ or *NRF2*^Over^ cells cultivated within selection medium. **g** LC-MS metabolic profiling showing the metabolic intermediates of glycolysis, PPP, TCA, and glutaminolysis. (*n* = 3 independent experiments; *t*-test, *P < 0.05; **P < 0.01; ***P < 0.001)

We next to further determine whether NRF2 activation plays a crucial role in up-regulating the Warburg effect. Compared with the Control^GFP^ cells, the *NRF2* overexpressing (*NRF2*^Over^) cells displayed the increased protein levels of glycolytic enzymes (Fig. 4a). In opposite, *NRF2* knockdown impaired glycolysis, including inhibiting the expression of glycolytic enzymes, reducing the glucose consumption ability, inhibiting the lactate level, and decreasing the GLUT3 positive cells (Figs. 4b–e and Figure S4a). Conversely, *NRF2* overexpression increased the lactate levels (Fig. 4f), which again supported our observation that NRF2 bound to the LDHA promoter of CICs by ChIP-Seq analyses (Fig. 3b). We next measured the glycolytic flux (extracellular acidification rate or ECAR) to further understand the effect of NRF2 activation on glycolytic activity. As shown in Figure S4b, the baseline ECAR was significantly increased in the *NRF2*^Over^ cells. We also found that NRF2 directly induced PDK1 expression (Figs. 3b and 4a) thus to inactivate PDH activity, as a result, to suppress the influx of glycolytic metabolites into the mitochondria^25^, ultimately, to lead to a decrease of ROS production^26^. In opposite, the sh*-NRF2* sphere cells showed more PDHA1 expression and red JC-1 aggregates than the Control cells (Fig. 4c and Figure S4c).

To further elucidate the mechanisms by which activated NRF2 reprogrammed glucose metabolism, the metabolomes of *NRF*2^Over^ cells or Control cells were determined by liquid chromatography-mass spectrometry metabolic profiling. Consistent with our previous analyses, the *NRF2*^Over^ cells showed increases in the levels of intermediate metabolites of glycolysis and non-oxidative PPP; however, the levels of the intermediate metabolites of TCA cycle were significantly decreased (Fig. 4g and Figure S4d). Based on these data, we further hypothesized that NRF2 activation enhancing the Warburg effect, which might be required to maintain the properties of CICs.

### NRF2 expression is required for CICs maintenance

**Fig. 5:**
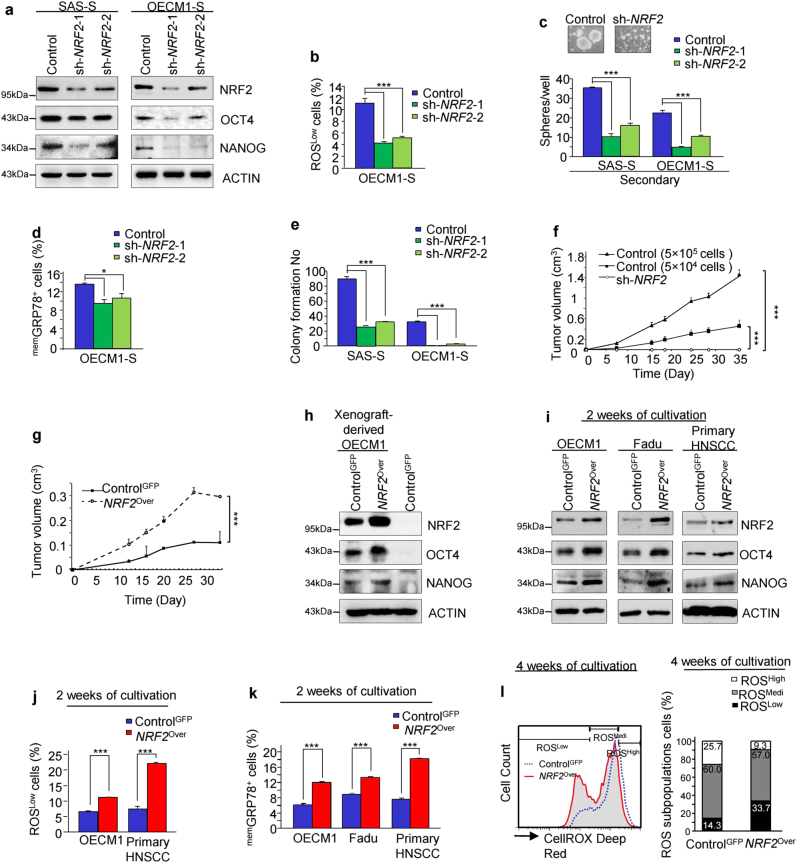
NRF2 maintains the CICs properties. **a** Immunoblots showing NRF2, OCT4, NANOG, and actin protein levels in the Control (sh-Luc) and sh*-NRF2* sphere cells. **b** Expression profiles of ROS^Low^ subpopulation cells in distinct lentivirus-infected sphere cells were determined via FACS. **c** Secondary spheres forming ability of sh*-NRF2* sphere cells. **d** The percentage of ^mem^GRP78-positive cells in the sh*-NRF2* sphere cells were determined via FACS. **e** Soft agar colony formation ability of sh*-NRF2* sphere cells (SAS-S (right panel), OECM1-S (left panel)). **f**–**g** Tumor growth curves of the sh-Luc or sh*-NRF2* SAS-S cells (**f**) and Control^GFP^ cells or *NRF2*^Over^ cells (**g**). Error bars correspond to SD (***P < 0.001). **h** NRF2, OCT4 and NANOG protein expression levels in xenografted tumors derived from Control^GFP^ cells and *NRF2*^Over^ cells or in *NRF2*^Over^ OECM1 cells. **i** NRF2, OCT4 and NANOG protein levels in HNSCC cells expressing GFP or *NRF2*-GFP cultivated in selection medium for 2 weeks. **j**–**k** The percentages of ROS^Low^ (**j**) and ^mem^GRP78 (**k**) cells in Control^GFP^ or *NRF2*^Over^ cells, grown in selection medium for 2 weeks, were determined via FACS. **l** The percentage of each subpopulation of ROS^Low^, ROS^Medi^ and ROS^High^ cells from the Control^GFP^ cells or *NRF2*^Over^ cells, grown in selection medium for 4 weeks, were determined via FACS. The results are presented as the mean ± SD of triplicate samples from three experiments (*P < 0.05; ***P < 0.001)

As shown in Figs. 3 and 4 that NRF2 mediated the CIC metabolism. Therefore, we further expected that NRF2 would regulate the stemness features of CICs. We first examined the endogenous NRF2 expression in HNSCC cancer cell lines. The endogenous NRF2 protein level was higher in SAS and OECM1 cells than in the other HNSCC cells (Figure S5a). Furthermore, human oral keratinocytes expressed the least amount of NRF2 protein among the examined cell lines (Figure S5a). As shown in Figure S5b, the sh*-NRF2* sphere cells did not maintain floating spheres but showed more attached epithelial-like cells. Furthermore, *NRF2* knockdown attenuated the expression of stemness markers (OCT4 and NANOG) (Fig. 5a). Since redox homeostasis could be mainly regulated by NRF2, we examined the ROS levels in sh*-NRF2* sphere cells. As expected, the percentage of ROS^Low^ cells was significantly reduced in sh*-NRF2* sphere cells, supporting the importance of NRF2 activation for ROS control (Fig. 5b). We have showed that the ^mem^Grp78^+^ cells possess enhanced stemness properties and tumorigenicity^18^. Accordingly, silencing *NRF2* expression in sphere cells markedly decreased their sphere-forming capacity (Fig. 5c and Figure S5c) and inhibited the amounts of cell surface Grp78 (^mem^GRP78^+^) and CD44^+^ cells^3, 27^ (Fig. 5d and Figure S5d). In contrast, the sh*-NRF2* sphere cells displayed enhanced differentiation and elevated apoptosis (Figures S5e and S5f). We also observed that the sh*-NRF2* sphere cells showed decreased anchorage-independent growth ability and migration (Fig. 5e and Figure S5g, S5h). Strikingly, Control sphere cells generated tumors in mice, whereas *NRF2*-knockdown sphere cells failed to form tumors (Fig. 5f and Figure S5i).

We further examined whether *NRF2* overexpression could promote their cancer-initiating capacity. The *NRF2*^Over^ (*NRF2*-overexpressing) cells displayed increased expression of stemness markers versus the control cells (Control^GFP^) (Figures S5j-S5m). Notably, *NRF2* overexpression also enhanced the tumorigenicity of OECM1 cells in vivo (Fig. 5g and Figure S5n). Empirically, we showed increased expression of stemness genes in xenograft-derived *NRF2*^Over^ cells compared with xenograft-derived Control^GFP^ cells (Fig. 5h). Additionally, microscopic images of control tumor specimens displayed pleomorphism and keratin whorls, suggestive of a differentiated cell phenotype, while the *NRF2*^Over^ specimens showed a more malignant cellular phenotype (Figure S5o).

We next sought to determine whether *NRF2* overexpression would promote the CIC properties when the HNSCC cells were cultivated within defined selection medium. As expected, the *NRF2*^Over^ cells showed an enhanced sphere-forming capacity (Figure S5p). Furthermore, more ROS^Low^ cells and increased expression levels of stemness markers were detected in the *NRF2*^Over^ cells than in the Control cells after cultivation in defined selection medium for 2 weeks (Fig. 5i–k and Figure S5q-S5s). Additionally, the *NRF2*^Over^ cells gave rise to a large increase in the number of ROS^Low^ cells but a decrease in the number of ROS^High^ cells compared with the control cells after a four-week cultivation (Fig. 5l). Thus, NRF2 activation not only promotes CIC properties but also facilitates an increase in the amount of ROS^Low^ cells.

### NRF2 up-regulates the expression of glycolytic enzymes and stemness markers by activating GRP78/PERK signaling in HN-CICs

**Fig. 6:**
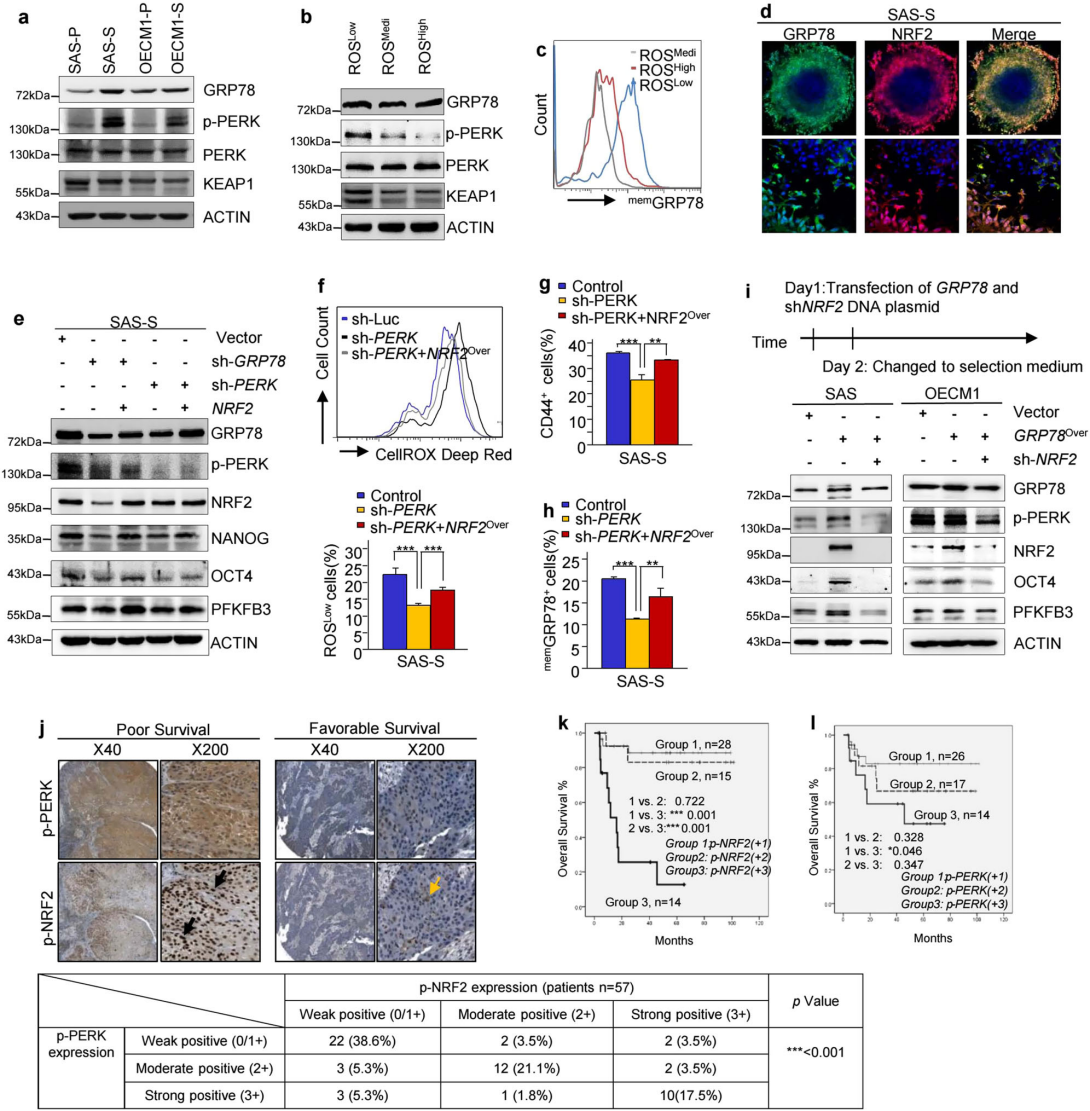
GRP78/p-PERK/NRF2 signaling induces the expression of glycolysis-related genes in CICs. **a**–**b** GRP78, p-PERK, PERK and KEAP1 protein levels in parental and sphere cells (**a**) or ROS^Low^, ROS^Medi^ and ROS^High^ cells (**b**). **c** FACS analyses showing the expression profiles of ^mem^GRP78^+^ cells in ROS^Low^, ROS^Medi^, and ROS^High^ cells, respectively. **d** Confocal IF staining of GRP78 and NRF2 in SAS-S cells. **e** Immunoblots showing the effects of *GRP78* knockdown (sh-*GRP78*), *PERK* knockdown (sh-*PERK*) and *NRF2* overexpression on GRP78/p-PERK/NRF2 signaling, NANOG, OCT4 and PFKFB3 expression in SAS-S cells. **f**–**h** Quantification of the ROS^Low^ (**f**), CD44 (**g**) or ^mem^GRP78 (**h**) positive SAS-S cells with *PERK* knockdown and/or *NRF2* overexpression. The data are presented as the mean ± SEM. **i** Overview of the transfection strategy. Effects of *GRP78* overexpression and/or *NRF2* knockdown on the activation of GRP78/p-PERK/NRF2 signaling, and expression of stemness proteins and glycolytic enzymes in SAS and OECM1 cells. **j** (Upper panel) IHC staining for p-NRF2 and p-PERK in HNSCC tumor tissues (p-NRF2-positive staining (black arrows: nucleus staining; yellow arrows: cytoplasmic staining)). (Lower panel) Fisher’s exact test showing the association according to p-NRF2 and p-PERK expression. **k**–**l** Kaplan–Meier analysis showing the overall survival of patients with HNSCC according to p-NRF2 (**k**) and p-PERK (**l**) protein expression

We next wanted to identify the molecular mechanism by which upregulated NRF2 activation to further reprogram the metabolism in HN-CICs. It has also been reported that NRF2 is directly activated by the Grp78/p-PERK signaling, which strongly correlates with chemotherapy resistance, tumor grade, and overall survival^28–31^. Therefore, we compared the expression profile of GRP78 and p-PERK proteins in sphere cells to that in parental cells. Accordingly, the protein levels of p-PERK were up-regulated in sphere or ROS^Low^ cells (Figs. 6a, b). Although the protein level of the GRP78 in ROS^Low^, ROS^High^, and ROS^Medi^ subpopulations of cells did not show a significant difference (Fig. 6b), the expression profile of ^mem^GRP78^+^ was significantly up-regulated in the ROS^Low^ cells than that in the ROS^High^ or ROS^Medi^ cells (Fig. 6c). Furthermore, immunofluorescent staining revealed co-expression of NRF2 with ^mem^GRP78 in the sphere cells (Fig. 6d). Together, the levels of the KEAP1 was not down-regulated in ROS^Low^ cells (Fig. 6b), suggesting that the NRF2 constitutive activation in ROS^Low^ cells is mediated through GRP78/p-PERK signaling but not by KEAP1 down-regulation.

Next, we wanted to assess GRP78/p-PERK inactivation in sphere cells to determine whether GRP78/p-PERK signaling controls NRF2 activation in CICs. As shown in Fig. 6e and Figure S6a, knockdown of *GRP78* or *PERK* diminished the protein levels of NRF2, stemness markers, and downstream targets. Conversely, *NRF2* overexpression partially restored the inhibitory effects of *GRP78* or *PERK* knockdown in sphere cells (Fig. 6e). As expected, *PERK* knockdown in sphere cells caused a significant reduction in the ROS^Low^ subpopulation cells and the amounts of CD44^+^ and ^mem^GRP78^+^ cells; again, this inhibitory effect of *PERK* knockdown was significantly reversed by *NRF2* overexpression (Figs. 6f–h and Figure S6b-S6d). Moreover, ectopic overexpression of *GRP78* not only up-regulated p-NRF2 and p-PERK expression but also enhanced the protein levels of glycolytic enzymes and stemness markers in HNSCC cells grown in defined selection medium (Fig. 6i). However, the *GRP78*^over^ cells which were co-transfected with sh*-NRF2* to deplete NRF2 expression showed decreased expression of glycolytic enzymes and stemness markers (Fig. 6i).

We next established the ontogeny of p-NRF2 or p-PERK expression in tissue microarrays from 57 resected HNSCC specimens to assess whether co-expression of p-NRF2 and p-PERK was associated with clinical prognosis. High p-PERK expression was positively correlated with nuclear NRF2 accumulation and was associated with a poor outcome (Fig. 6j). Furthermore, the survival of patients with HNSCC was negatively correlated with the expression of p-NRF2 and p-PERK in the Kaplan-Meier survival analysis (Figs. 6k, l).

**Fig. 7:**
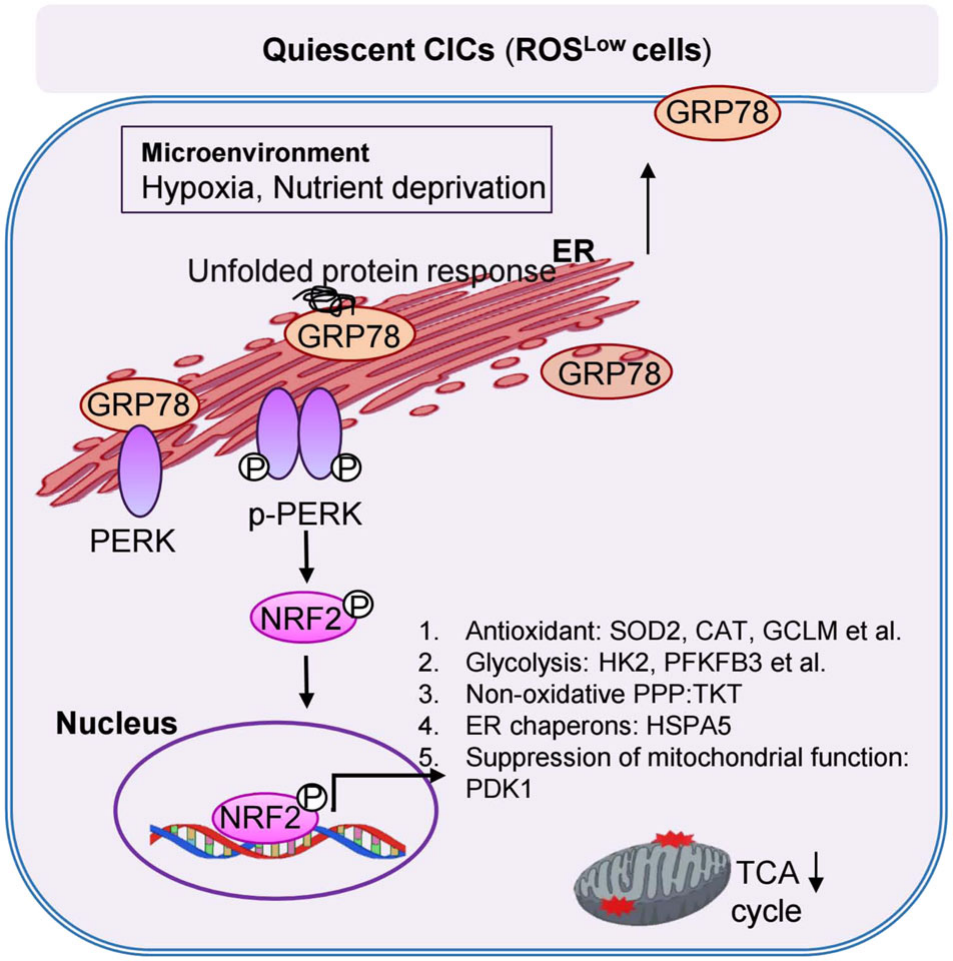
Schematic model for the role of GRP78/p-PERK/NRF2 signaling in up-regulating stemness properties, antioxidant activity, ER stress and metabolic signatures in ROS^Low^ cells. Heterogeneity of CICs is demonstrated with respect to redox status. NRF2 activation directly increases glycolysis and non-oxidative PPP activity through GRP78-PERK signaling activation in ROS^Low^ cells

In summary, up-regulation of the GRP78/p-PERK pathway increases the expression of NRF2 and enables NRF2 to promote the glycolytic activity and antioxidant properties, which can promote stemness properties (Fig. 7).

## Discussion

ROS status is associated with metabolic reprogramming of both stem cells and CICs, which results in adaptations to microenvironmental stress^8^. Activation of glycolysis and suppression of mitochondrial metabolite flux are an effective strategy for stem cell maintenance^32^. In fact, the promotion of “Warburg effect” results in several metabolic benefits for CICs, such as reduced oxygen dependence and ROS production, maintenance of ATP production in response to hypoxic stress and increased production of glycolytic intermediates for biosynthesis^6, 33, 34^. Because mitochondrial OXPHOS is a major source of ROS production and has been intimately linked to stem cell proliferation and differentiation^35, 36^, the quiescent ROS^Low^ CICs may benefit from using glycolysis, as they successfully survive and maintain their quiescent state and stemness properties in response to the stress from the tumor microenvironment.

Distinct subsets of CICs with different ROS levels display different metabolic activities, which are associated with the NRF2-targeted genes (Fig. 3). Indeed, NRF2 activated the glycolysis-related genes in quiescent ROS^Low^ cells, which was advantageous for maintaining low ROS levels within the CIC niche. In contrast, NRF2 activated the expression of enzymes required for oxidative PPP and glutathione synthesis in ROS^High^ cells (Fig. 3a). Our observations are consistent with others’ study showing that NRF2 directly increases the expression of enzymes involved in the PPP and glutamine synthesis in cancer cells to facilitate increased proliferation^24^. Overall, NRF2 activation would affect various metabolic pathways within different tumor subtypes to generate tumor heterogeneity, consistent with the observation that NRF2 may play distinct roles in stem cells because it activates various target genes^12, 37^.

NRF2 may regulate over 100 genes within the promoter regions^38^. Intriguingly, we observed a decreasing trend in the expression levels of some NRF2-regulated canonical genes in sphere cells in the microarray. Additionally, our ChIP-Seq analyses revealed that sphere cells were NRF2 up-regulated but did not trans-activate typical NRF2 target gene NQO1. Accordingly, sphere cells displayed decreased expression of NQO1 (Figure S2c), suggesting that NRF2 may regulate genes other than classic cytoprotective genes in CICs.

ROS-independent NRF2 activation as a major effector of PERK-mediated cancer cell and stem cells survival has been recognized^28, 39, 40^. The potential contribution of global translation arrest control through p-PERK regulation of eIF2α cannot be excluded^41^. In addition, we also uncovered that the EIF2 signaling was upregulated in ROS^Low^ and sphere cells (Figure S1h). These data lead us to hypothesize that active PERK-NRF2/EIF2 signaling may also play an additional role to maintain the quiescent state of ROS^Low^ cells. Upon ER stress, GRP78 activates PERK/NRF2 signaling to promote cancer cell survival^42^. Stress may promote the re-localization of GRP78 to the cell membrane, which acts as a critical regulator of oncogenic signaling^18, 43, 44^. Here, overexpression of GRP78 in cells grown in defined selection medium promoted the re-localization of GRP78 to the membrane (Figure S6f), which activated both PERK and NRF2 and increased the stemness properties and glycolytic activity (Fig. 6 and Figure S6e). Consistent with our results, GRP78 is associated with modulation of the glycolysis pathway in stem cells^31^. Interestingly, GRP78 was one of the direct targets of NRF2 through NRF2 ChIP-Seq analysis of sphere cells (Figure S3f). NRF2 overexpression could restore GRP78 expression in GRP78-knockdown sphere cells (Fig. 6e and Figure S6a). Thus, the positive GRP78/p-PERK/NRF2 feedback loop in ROS^Low^ cells appears to be a major mechanism for constitutively maintaining lower ROS levels.

In summary, this is the first finding that a positive regulatory loop of GRP78/p-PERK/NRF2 signaling mediates the Warburg effect and stemness properties of CICs in a ROS-independent manner. NRF2 signaling regulated the levels of different metabolic activities to adapt to the environment and to modulate cell behaviors in CICs containing different ROS levels. Therefore, the molecular mechanisms underlying these distinct metabolic pathways may reveal strategies for tumor-specific therapy.

## Materials & Methods

### Chromatin immunoprecipitation (ChIP) sequencing

For ChIP-Seq libraries of human SAS sphere cells or parental cells, input DNA has been used as the control. Full details are given in the in the Supplementary Material and Methods.

### Fluorescent glucose uptake assay

Following 30 min of glucose starvation, stable *NRF2*-knockdown sphere cells were incubated in the presence of a fluorescent glucose analog, 2-[*N*-(7-nitrobenz-2-oxa-1,3-diaxol-4-yl)amino]-2-deoxyglucose (2-NBDG) for 30 min at 37 °C. The cells were then resuspended in PBS containing 2% FBS and stained with propidium iodide. The 2-NBDG fluorescence was measured using a FACScanto flow cytometer (Becton Dickinson).

### Patient population and immunohistochemistry

Between 2004 and 2006, 57 patients with operable head and neck cancer, without histories of radiation therapy or chemotherapy, underwent surgical treatment at Taipei Veterans General Hospital (Taipei, Taiwan) (IRB No.1000075). This study was approved and accorded with the institutional review board and ethics committee of Taipei Veterans General Hospital and National Yang Ming University. The deparaffinization, rehydration, antigen retrieval, antibody hybridization, visualization, and grading were performed as described previously^18, 45^.

### Statistical analysis

The data are presented as the mean ± SD of three independent experiments. Continuous variables between groups were compared with the independent Student’s *t*-test or ANOVA. We used the Statistical Package for the Social Sciences software (version 13.0) (SPSS, Inc., Chicago, IL) to generate the Kaplan-Meier survival curves and to determine statistical significance. *P* < 0.05 was considered a significant difference for all of the tests.

## Electronic supplementary material

Supplementary Figures

Supplementary materials and methods

Supplemental table
