# Supplementary material for: ROS-independent ER stress-mediated NRF2 activation promotes warburg effect to maintain stemness-associated properties of cancer-initiating cells
Source: Cell Death Dis. 2018 Feb 7;9(2):194. doi: 10.1038/s41419-017-0250-x (PMC5833380; doi:10.1038/s41419-017-0250-x)
Supplement: Supplementary file 1 — Supplementary Figures [file 41419_2017_250_MOESM1_ESM.pdf]

Supplementary Figure 1. Chang et al.

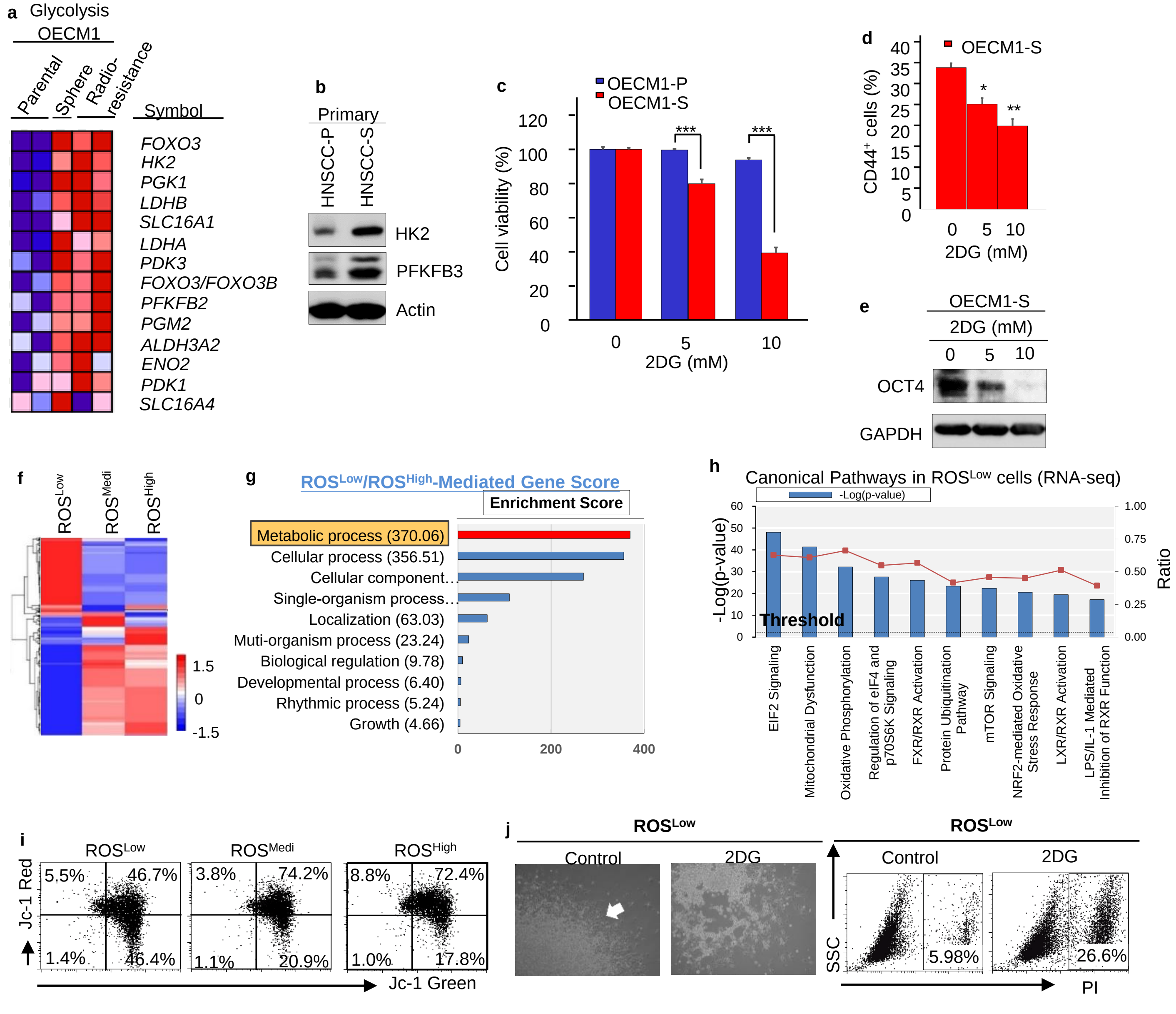

Supplementary Figure 1. ROS^{Low} CICs relying on Warburg effect

(a) Heatmap depicting expression of genes encoding glycolytic pathway in parental, sphere cells and radio-resistance cells. (b) Immunoblot detecting the glycolytic enzymes in primary parental and sphere cells. (c) Parental cells (OECM1-P) or sphere cells (OECM1-S) were treated with 0, 5 or 10 mM of 2DG for 48 hr, afterward, stained with propidium iodide (PI) and then examined by flow cytometry. The PI-negative cells were recorded as viable cells (***p < 0.001). (d) Expression profile of CD44-positive cells of 2-DG treated sphere cells was analyzed by flow cytometry. The bar graph shows quantification of CD44-positive cells. (e) Immunoblots showing the expression of OCT4 and GAPDH in sphere cells with or without 2-DG treatments. (f) Heatmap and dendrogram show significant gene expression patterns (P<0.05, 1.5-fold change) among ROS^{Low}, ROS^{Medi} and ROS^{High} cells by RNA-Seq analyses. (g) Summary of the scores for the major biological processes in the GO categories observed in ROS^{Low} cells using RNA-Seq analysis. These scores reflect absolute gene numbers in each major category. The abscissa represents the number of CDSs assigned to the corresponding category.) (h) Top ten canonical pathways identified in ROS^{Low} cells using RNA-Seq analysis. (i) The mitochondrial membrane potentials (Δψ, red/green ratios) of the sorted ROS^{Low}, ROS^{Medi} and ROS^{High} cells were determined using JC-1 staining followed by FACS. (j) ROS^{Low} cells were sorted using DCFDA staining from SAS sphere cells. At day 21, the cells were treated with 2-DG, for 72 hours, afterward, stained with propidium iodide (PI) and then examined by flow cytometry. Representative images of 2-DG induced differentiation were shown.

Supplementary Figure 2. Chang et al.

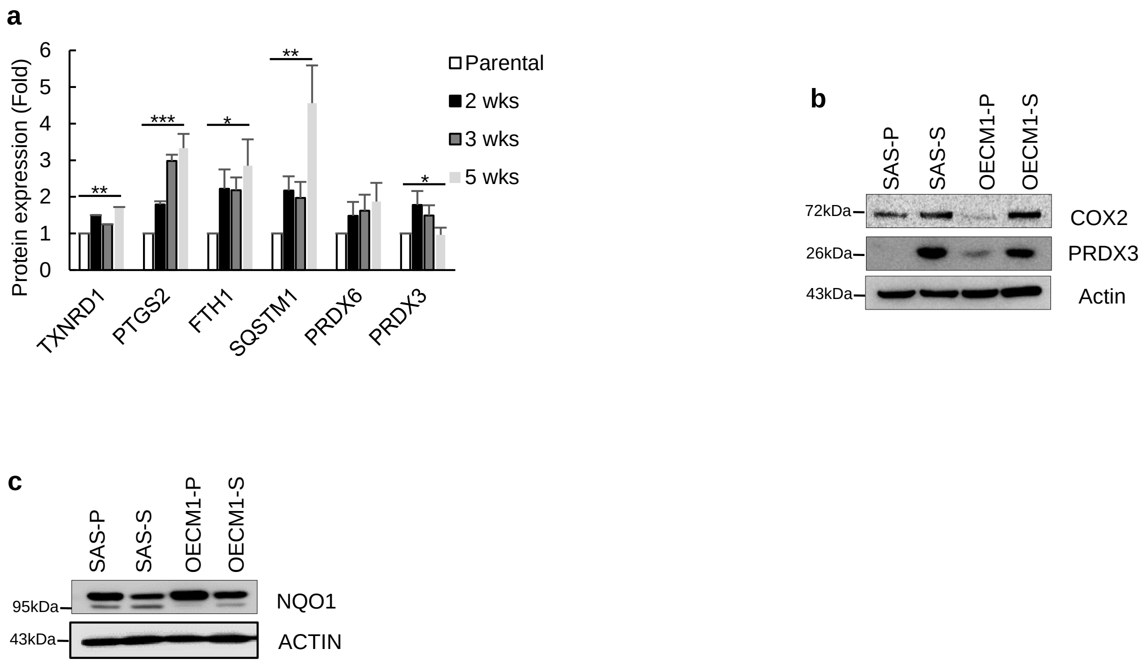

Supplementary Figure 2. NRF2 target genes are highly expressed in SAS sphere cells compared to parental cells
(a) Protein levels of NRF2 targeting genes in SAS sphere and parental cells membrane fractions. P-value, ANOVA test. * indicates $P<0.05$; ** indicates $P<0.01$; *** indicates $P<0.001$. (b) Immunoblots showing COX2, PRDX3 and Actin protein levels in parental and sphere cells. (c) Immunoblots showing NQO1 and Actin protein levels in parental and sphere cells.

Supplementary Figure 3. Chang et al.

a

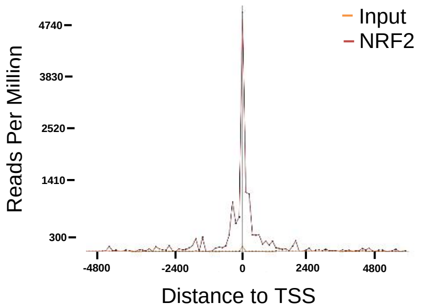

b

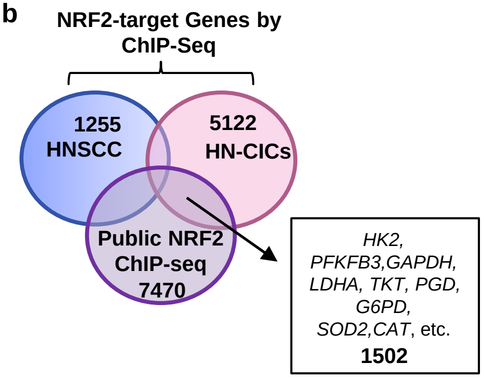

c

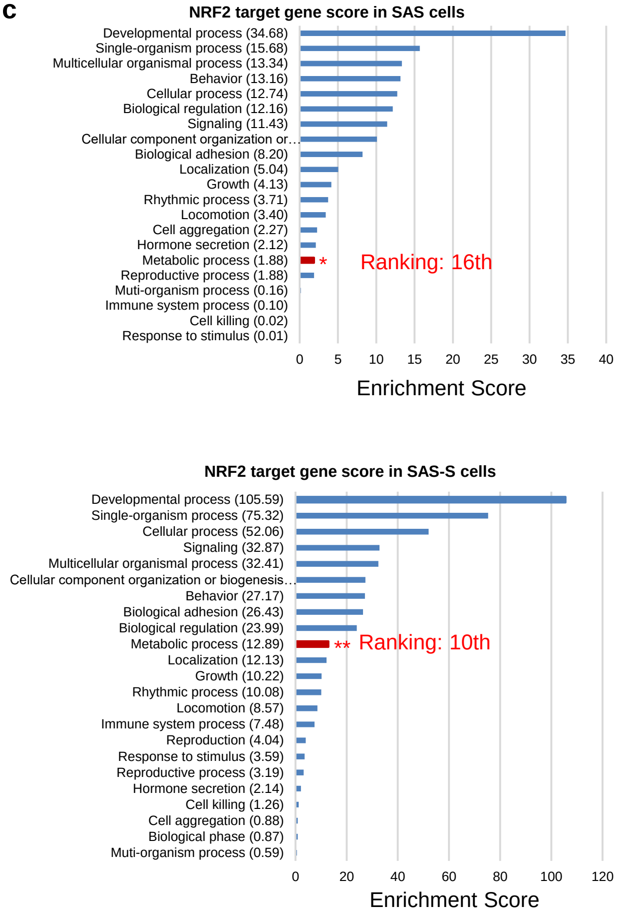

d Fold enrichment(NRF2/inpu)

	SAS	SAS-S	Distance to TSS
HK2	0	54.1685	1246
PFKFB3	0	63.4459	-1105
GAPDH	0	60.7154	0
PGK1	77.7368	135.888	0
ENO1	0	49.8799	-20
ENO2	95.4396	87.0409	120
LDHA	0	67.0875	0
PDK1	127.047	62.7133	57
G6PD	0	48.7325	727
PGLS	0	56.0293	0
PGD	0	45.1404	587
TKT	0	47.7627	-4469
GLS	0	92.3563	-250
GCLM	0	87.7855	99

Supplementary Figure 3. Chang et al.

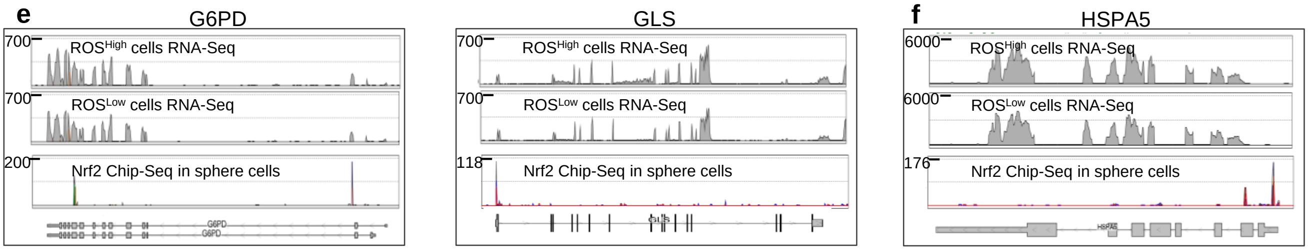

Supplementary Figure 3. HN-CICs Displaying Distinct Metabolic Features compared with HNSCC cells by activation of NRF2
(a) Distribution of NRF2 and input ChIP-seq peaks around the TSS of genes in SAS-S cells. (b) Venn diagram depicting the overlap genes of NRF2-bound genes in SAS cells (blue), sphere cells (pink) and the public NRF2 ChIP-seq data (purple). (c) Gene ontology analyses classifying the categories of major biological process upon NRF2-regulated genes. These data score reflects absolute gene numbers in each major category. The abscissa represents the number of CDSs assigned to the corresponding category. P-value, Fisher's exact test. * indicates P<0.05; ** indicates P<0.01. (d) CHIP-seq fold-enrichment signal of NRF2 in SAS parental and sphere cells, assessing the binding of glutamate, PPP, and glycolysis. (e,f) ChIP-Seq tracks of NRF2 in SAS sphere cells, assessing the binding of G6PD, GLS (e) and HSPA5 (f). Location of ChIP-Seq peaks that were identified by using Strand NGS software. RNA-Seq traces (UCSC genome browser) for each gene in ROS^{Low} cells and ROS^{High} cells.

Supplementary Figure 4. Chang et al.

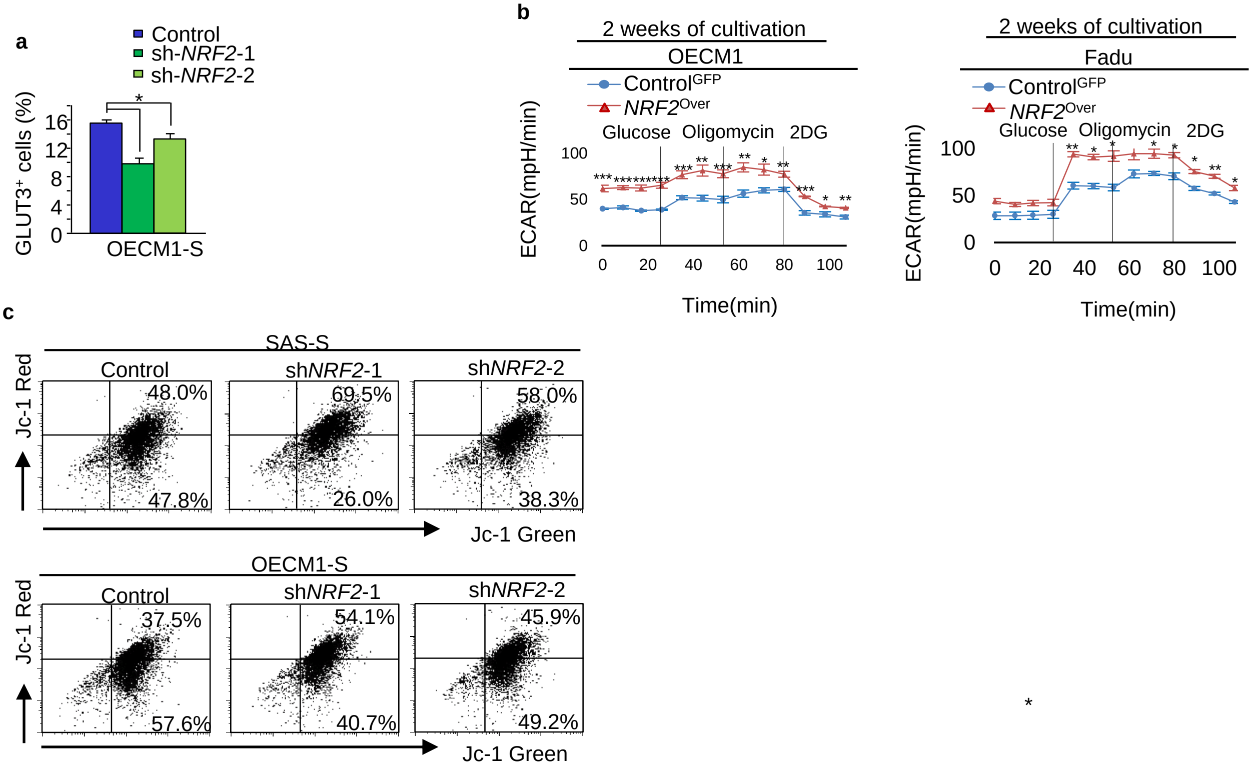

*

d

Fadu cells

Pathway		Fold (NRF2 / GFP)	P value
Glycolysis	glucose	1.02	0.9114
	glucose 6-phosphate / fructose 6-phosphate	1.28	0.0188
	fructose 1,6-bisphosphate	2.57	0.0229
	glyceraldehyde 3-phosphate / dihydroxyacetone phosphate	0.91	0.0279
	pyruvate	0.97	0.7837
TCA cycle	citrate / isocitrate	0.59	0.0092
	alpha-ketoglutarate	0.64	0.0460
	fumarate	0.48	0.0024
	malate	0.49	0.0024
PPP	6-phosphogluconate	0.81	0.0043
	ribose 5-phosphate / Ribulose 5-phosphate / xylulose 5-phosphate	1.24	0.0291
	erythrose 4-phosphate	1.12	0.8061
	sedoheptulose 7-phosphate	2.12	5.50E-04
	NADPH	0.52	0.0153
	NADP+	0.7	0.1985
Glutathione	GSH	0.97	0.567
	Glutamate	0.8	0.0238

OECM1 cells

Pathway		Fold (NRF2 / GFP)	P value
Glycolysis	glucose	1.62	6.30E-05
	glucose 6-phosphate / fructose 6-phosphate	0.92	0.300
	fructose 1,6-bisphosphate	0.77	0.118
	glyceraldehyde 3-phosphate / dihydroxyacetone phosphate	1.07	0.408
	pyruvate	1.01	0.872
TCA cycle	citrate / isocitrate **	1.37	0.000
	alpha-ketoglutarate	1.02	0.889
	fumarate	0.81	0.061
	malate	0.78	0.069
PPP	6-phosphogluconate	0.66	0.232
	ribose 5-phosphate / ribulose 5-phosphate / xylulose 5-phosphate	0.87	0.209
	erythrose 4-phosphate	0.70	0.153
	sedoheptulose 7-phosphate	0.99	0.949
	NADPH	0.63	0.483
	NADP+	0.38	0.246
Glutathione	GSH	1.92	0.0039
	Glutamate	0.95	0.3980

Supplementary Figure 4. f NRF2 mediated glycolytic pathway and mitochondrial membrane potential in sphere cells
(a) The expression profile of GLUT3-positive cells in the sh-*NRF2* sphere cells were determined via FACS analyses. (b) After treatment with indicated inhibitors, extracellular acidification rate (ECAR) were examined in Control ^{GFP} and NRF2^{Over} cells grown in selection medium for 2 weeks. ECAR was measured by Seahorse system. Traces shown are representative of at least two independent experiments in which each point represents the mean of three replicates (*, P < 0.05; **, P < 0.01; ***, P < 0.001). (c) Mitochondrial membrane potential in sh-NRF2 and control sh-Luc sphere cells was evaluated by JC-1 staining followed by FACS analysis. (d) Summary of metabolic changes in NRF2^{over} cells as compared to that of the Control^{GFP} cells using mass spectrometry

Supplementary Figure 5. Chang et al.

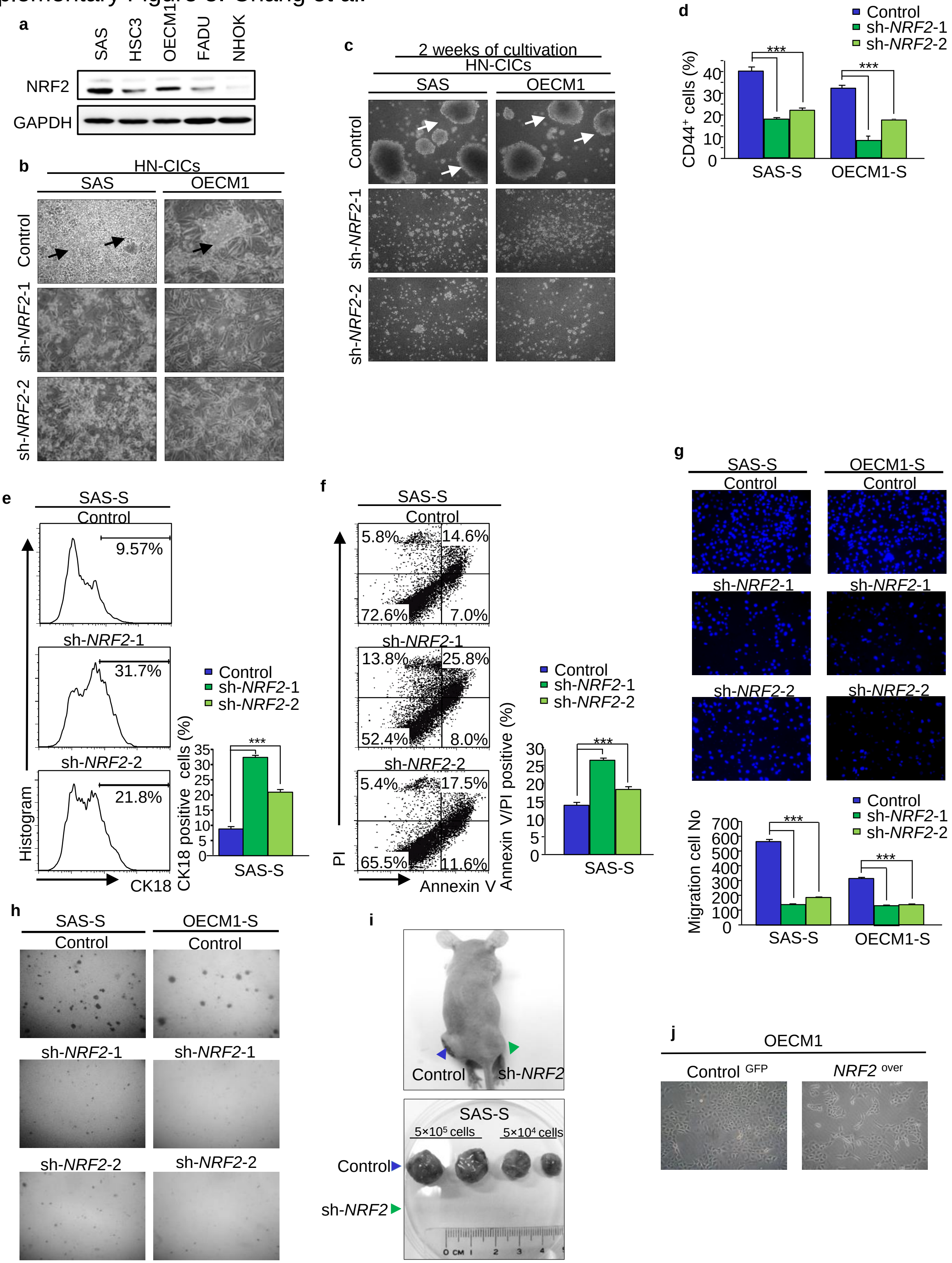

Supplementary Figure 5. Chang et al.

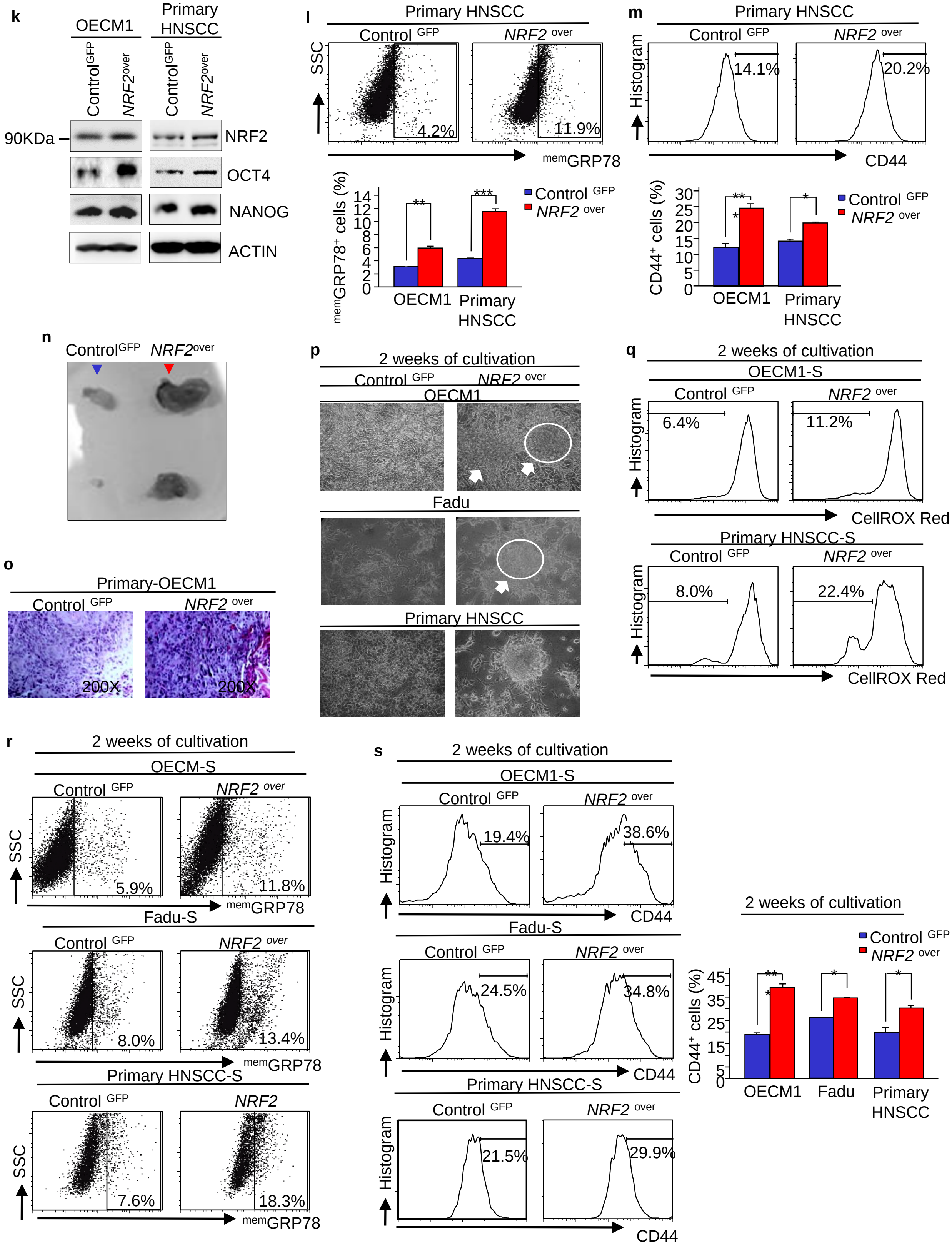

Supplementary Figure 5. Role of NRF2 in regulating cancer stem cell properties

(a) Crude extracted proteins were prepared from HNSCC cells and human oral keratinocytes (NHOK). The expression level of NRF2 protein of the crude extracted proteins was determined by immunoblot assay. (b) Sphere cells were first infected with sh-*NRF2*-1, sh-*NRF2*-2 or sh-Luc lentivirus for 2 days, and then further cultivated under the serum-free defined selection medium. The morphology of *NRF2*-knockdown sphere cells was observed by photograph. Arrows indicated the sphere cells. (c) The images showing the secondary sphere forming ability of SAS or OECM1 sphere cells with the *NRF2* knockdown. (d) The numbers of CD44-positive cells in the sh-*NRF2* sphere cells were determined via FACS analyses. (e-f) FACS analyses showing the percentages of CK18 (e) or Annexin V (f) positive staining cells of sphere cells infected with sh-*NRF2*-1, sh-*NRF2*-2 or sh-Luc lentivirus. The experiments were repeated three times, and the representative results were shown. Results are means ± SD (***, $p < 0.001$). (g) Effect of *NRF2* Knockdown on cell migration capability was analyzed, and images were obtained by phase contrast microscopy. (h) Microscopic images showing the anchorage-independent growth ability of sphere cell with *NRF2* down-regulation. (i) Images of the tumor growth in nude mice injected with sh-Luc and sh-*NRF2* SAS spheres (upper panel); the dissected tumor tissues from the recipient mice (lower panel (blue arrows: sh-Luc sphere cells; green arrows: sh-*NRF*2 sphere cells). (j) Morphological difference between $Control^{GFP}$ or $NRF2^{over}$ cells derived from OECM1 cells. ($Control^{GFP}$ cells (overexpressing GFP); $NRF2^{over}$ cells (overexpressing both the GFP and *NRF2* proteins)). (k) Immunoblots showing NRF2, OCT4, NANOG and actin protein expression in OECM1 cells and primary HNSCC cells stably overexpressing *NRF2*. L-M, The expression profile of $^{mem}GRP78$ (l) or CD44 (m) of $Control^{GFP}$ or $NRF2^{over}$ cells was assessed, respectively, by flow cytometry. (n) $Control^{GFP}$ cells or $NRF2^{over}$ cells derived from OECM1 cells were subcutaneously injected into nude mice. Images of the dissected tumors were captured on day 34. (o) Histological analysis of primary xenografts generated from $Control^{GFP}$ or $NRF2^{over}$ cells, respectively. Tumor sections were stained with H&E. (p) Representative images of sphere formation efficiency from $Control^{GFP}$ or $NRF2^{over}$ cells (OECM1 cells, Fadu cells and primary HNSCC) cultivated under serum free medium for 2 weeks. (q) Single cell suspensions of $Control^{GFP}$ or $NRF2^{over}$ cells, cultivated in selection medium for 2 weeks, then, were stained with CellROX Red dye. The intracellular low level of ROS in $Control^{GFP}$ or $NRF2^{over}$ cells, grown in selection medium for 2 weeks, were determined via FACS. (r) $^{mem}GRP78$ expression in $Control^{GFP}$ or $NRF2^{over}$ cells grown in selection medium for 2 weeks were analyzed by flow cytometry. (s) Cell surface CD44 expression in $Control^{GFP}$ or $NRF2^{over}$ cells grown in selection medium for 2 weeks were analyzed by flow cytometry.

Supplementary Figure 6. Chang et al.

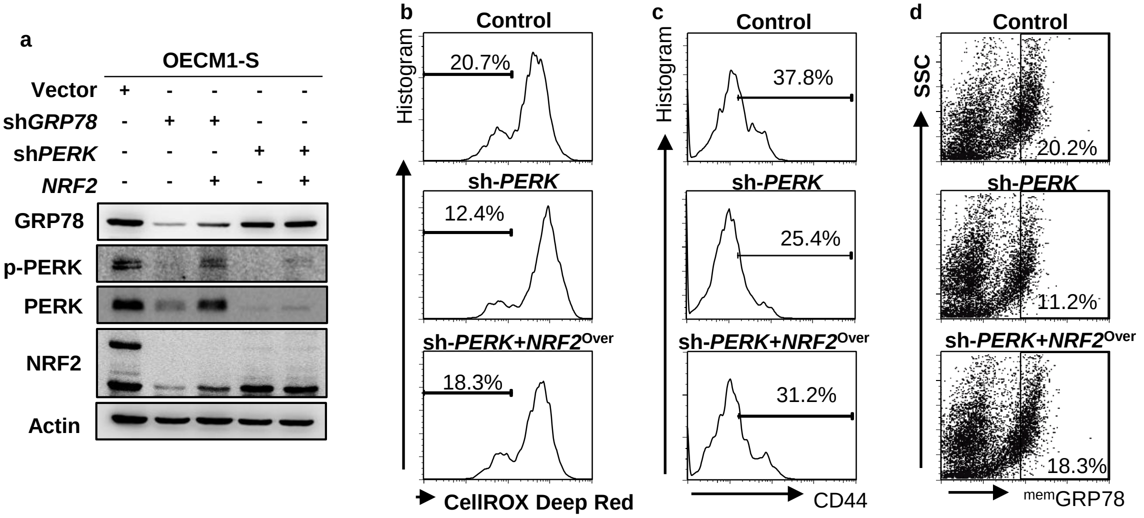

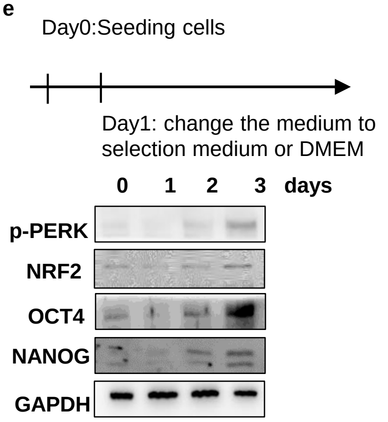

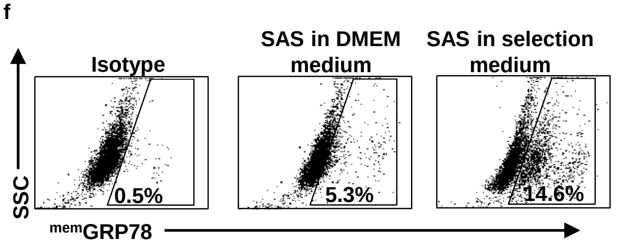

Supplementary Figure 6. NRF2 elevating expression of cancer stem cell marker upon activating GRP78/p-PERK signaling in HN-CICs
(a) Immunoblots showing the expression of GRP78, p-PERK, PERK, NRF2 and Actin in OECM1 sphere cells first transduced with lentiviruses (control vector, sh*GRP78*, sh*PERK*), and/or further infected with lentiviruses expressing *NRF2*. B-D, The percentages of ROS^{Low} (b), CD44 (c) and ^{mem}GRP78 (d) positive staining cells in *PERK* knockdown or/and *NRF2* overexpressing sphere cells were examined by FACS analysis, respectively. (e) An overview of the experimental strategy. Total proteins were prepared from cells cultivated with selection medium for 3 days and analyzed by immunoblotting against anti-p-PERK, anti-NRF2, anti-OCT4, anti-NANOG or anti-GAPDH antibodies as indicated. (f) The percentages of ^{mem}GRP78 positive staining cells cultivated under different conditione medium were examined by flow cytometry analysis.
