# Supplementary material for: ROS-independent ER stress-mediated NRF2 activation promotes warburg effect to maintain stemness-associated properties of cancer-initiating cells
Source: Cell Death Dis. 2018 Feb 7;9(2):194. doi: 10.1038/s41419-017-0250-x (PMC5833380; doi:10.1038/s41419-017-0250-x)
Supplement: Supplementary file 2 — Supplementary materials and methods [file 41419_2017_250_MOESM2_ESM.docx]

**Supplemental Materials and Methods**

Cell lines

The human head and neck cancer cell lines, Homo sapiens pharynx squamous cell lines

Fadu and human oral squamous cell carcinoma cell lines HSC-3 were originally from ATCC and maintained using recommended culture conditions. The human tongue carcinoma cell lines SAS were obtained from the Japanese Collection of Research Bioresources (Tokyo, Japan) ^1^ were cultured in DMEM medium with 10% FBS (Gibco BRL and Life Technologies) and 1% L-glutamine. The human gingival squamous carcinoma cells (OECM-1) were provided from by Dr. Ching-Liang Meng (National Defense Medical College, Taipei, Taiwan) and grown in RPMI 1640 medium supplemented with 10% FBS. The primary cultures of HNSCC cells (a gift from Dr. Muh-Hwa Yang, National Yang-Ming University) were cultured in RPMI 1640 supplemented with 10% FBS. Cells were cultured at 37°C containing 5% CO_2_.

Harvesting of HN-CICs by Sphere formation assay

Cells were seeded at a density of 7.5×10^4^ live cells/10-mm dish in defined selection medium [DMEM/F12 medium (GIBCO) containing with N2 supplement (GIBCO), 10 ng/mL human recombinant basic fibroblast growth factor-basic (FGF) and 10 ng/mL Epidermal Growth Factor (EGF) (R&D Systems, Minneapolis, MN)]. The medium was changed every other day until the sphere formation was achieved by approximately 4 weeks.^2^

For investigating the capability of secondary spheres formation, the primary spheres were trypsinized to a single cell suspension and then transferred to 6-well low-attachment plates at a concentration of 1×10^4^ cells per well in defined selection medium. After 14 days, the number of spheres larger than 50 μm in diameter was counted.

Establish radiation resistant cells

The OECM1 cells were exposed to repeated g-ray radiation (ionizing irradiation), and after g-ray radiation and repeat three times, a radioresistant cell was obtained. The g-ray radiation was delivered by Theratronic cobalt unit T-1000 at a dose rate of 1.1 Gy/min (SSD = 57.5 cm).^3^

ROS assay and cell sorting

For intracellular ROS analysis, cells were resuspended in PBS containing 2% FBS at a density of 10^6^ cells/mL and loaded with 10 μM DCF-DA (Thermo Fisher Scientific) or 2.5 μM CellROX Deep Red (Thermo Fisher Scientific), incubated at 37°C for 30 min. An oxidation-insensitive analog of Carboxy-DCFDA (Thermo Fisher Scientific) staining was used as a control to detect the probe uptake, probe efflux, and nonspecific probe activation. Cells were washed and resuspended in PBS containing 10 uM propidium iodide (PI) solution for analysis or sorted on a FACSAria cell sorter (Becton Dickinson). Each experiment, dead cells were excluded by PI staining. Isolation of ROS^Low^, ROS^Medi^, and ROS^High^ cells was performed as described previously.^4^

Fractionations of nuclear and cytosolic lysates

Parental and sphere cells were incubated in cytosolic lysis buffer [20 mM Hepes pH 7.9, 10 mM KCl, 10 mM NaF, 0.5% NP-40, 2 mM MgCl_2_ and a protease inhibitor tablet (Roche)] for 10 min.  Protein samples from cytosolic and nuclear fractions were separated by centrifugation at 4000 × rpm for 5 min at 4 °C. The supernatant was used as cytoplasmic fraction. The pellet was resuspended in nuclear lysis buffer (150 mM NaCl, 1 mM EDTA, 20mM Tris-HCl, 0.5% NP-40, 10mM NaF and a protease inhibitor tablet) and vortex for 60s. Nuclei extract was obtained from the supernatant after centrifuging at 1, 2000 rpm for 20 min.

Plasmid Construction

The cDNA fragments encoding full-length Nrf2 was generated by PCR-mediated ligation and purified then cloned into the pCDH-MCS1-EF1-copGFP vector from Biosettia Inc. (Biosettia, San Diego, CA). Lentivirus production was performed by transfection of plasmid DNA mixture with lentivector plus helper plasmids (VSVG and Gag-Pol) into 293T cells using polyjet (SignaGen Laboratories, Rockville, MD, USA) according to the manufacturer's protocol. Supernatants were collected 48 hours after transfection and then were filtered. To generate the stable cell lines, Sub-confluent cells were infected with lentivirus in the presence of 8 μg/ml polybrene (Sigma-Aldrich, St Louis, MO, USA). The green fluorescence protein (GFP), which was co-expressed in lentiviral-infected cells, was served as a selection marker to indicate the successfully infected HNSCC. Stable Nrf2-overexpressing HNSCC cell lines were further purified by cell sorting with GFP positive cells (Data not were shown). The pCDH1-MCS1-EF1-copGFP empty vector alone is utilized for experimental control.

Grp78 plasmid (pCMV-GRP78) was kindly provided by Dr. Ann-Joy Cheng, Chang (Gung University, Taipei, Taiwan). pCMV-GRP78 cDNA was introduced HNSCCs transiently by transfection. All plasmids used were confirmed by sequencing.

shRNA Knockdown

The lentiviral shRNA plasmids for the knockdown of Nrf2, Grp78, PERK, HK2 or PFKFB3 were obtained from the National RNAi Core Facility (Academia Sinica, Taiwan). The method of lentivirus production and cell infection was performed using the manufacturer’s protocol. Stable shRNA-expressing cell lines were further selected by puromycin (1-2 µg/mL) selection. The detailed target sequences are provided in Table S1.

Immunofluorescence staining

In brief, cells plated onto glass coverslips were fixed with 4% paraformaldehyde and then washed with PBS three times. The cells were incubated with primary antibodies [Grp78 (BD Transduction Laboratories™)]. Cells then were permeabilized with 0.1% Triton X-100/PBS for 10 minutes. Consequently, the cells were incubated with primary antibodies [Nrf2 (Santa Cruz Biotechnology, Santa Cruz, CA)]. After PBS washing (three times, 5 min each), cells were further probed with FITC or PE-tagged secondary antibodies. DAPI was used for nuclear staining. The fluorescence intensity was recorded by inverted fluorescence microscope equipped with CCD camera (Zeiss LSM880 with AiryScan) and the percentage of fluorescence signal per photographed field was analyzed by LSM880 Zen Blue Software.

Cell anchorage-independent growth assay

Each well of a six-well culture dish was coated with 2 ml of bottom agar mixture [DMEM containing 10% FBS and 0.6% agar (Sigma-Aldrich)]. After the bottom layer was solidified, 1×10^4^ virus-infected sphere cells were cultured in 2 ml of a top agar-medium mixture (DMEM containing 10% FBS and 0.3% agar), and the dishes were incubated for 2 weeks at 37^o^C. Subsequently, the plates were stained with 0.005% crystal violet, and then counted the colonies. The number of total colonies was counted under a microscope. Each experiment was done in triplicate.

In vitro cell migration assay

For transwell migration assays, 2x10^5^ cells suspended in medium with lower serum (0.5% FBS) were plated into the top chamber of a transwell (Corning, Acton, MA) with a non-coated membrane (8.0 mm pore size). And then medium supplemented with higher serum (10% FBS) was added as a chemoattractant in the lower chamber. The cells then incubated at 37 °C for 24 h. Subsequently, the cells on the lower surface of the membrane were removed with a cotton swab and cells on the lower surface of the Transwell were stained with Hoechst 33258 (Sigma-Aldrich) to show the nuclei. The number of fluorescence cells was counted under a microscope. Each experiment was done in triplicate.

RNA Preparation, quantitative RT-PCR, and RNA-Seq.

Total RNA was prepared using TRIzol reagent (Invitrogen), according to the manufacturer's instructions. cDNA synthesis was performed using SuperScript­III reverse transcriptase (Invitrogen) with random hexamer primers. Quantitative real-time RT-PCR was performed using the StepOne-Plus real-time PCR system (Applied Biosystems) using a Fast SYBR® Green master mix (Applied Biosystems). The relative expression levels of mRNA were normalized to actin mRNA levels. The primer sequences used for the RT-PCR are listed in Table S2.

RNA-Seq libraries were sequenced paired-end (100 bp) on a HiSeq2500 (Illumina). Base calling was conducted by Illumina’s software packages RTA1.17.21.3. Raw reads were processed using the Illumina CAS (v. 1.8.2) to filter out the low-quality reads. RNA-Seq reads were aligned to the GRCh38 genome assembly using CLC Genomics Workbench v8.0. The accession number of our subject is GSE97197.

Cellular Extracellular acidification rate

Extracellular acidification rate (ECAR) was measured by Seahorse system. A total of 100,000 stable Nrf2-overexpression sphere cells were plated in Seahorse XF24 plates. One hour before measurement, the culture medium was replaced with defined serum-free selection medium without glucose. ECAR upon sequential injection three metabolic inhibitors into the medium as a substrate during detection: Glucose (10 mM), followed by ATP synthase inhibitor oligomycin (1μM), followed by 2-deoxy-D-glucose (2-DG) (100 mM). Data were analyzed by Seahorse XF24. Results were normalized to cell number.

Metabolite extraction and LC-MS metabolic profiling

Cell metabolites were extracted by adding 1ml of pure methanol to the cell body, mixed by proper vortex, and storage at -80^o^C. The debris was removed by centrifugation at 13000g for 10min, then the supernatants were transferred to another microcentrifuge tubes and dried in a vacuum sample concentrator at room temperature for 2 hours. The harvested metabolites were weighed and reconstituted with pure water to the concentration of 7.5 ug total metabolites/ ul water.

For LC-MS analysis, a UPLC system (Waters, USA) equipped with a binary solvent delivery system and an autosampler was used. The cell extracts were injected into an Acquity UPLC BEH C18 column (2.1 × 100 mm, 1.7 μm; Waters) and equilibrated with water containing 0.1% ammonium hydroxide. The samples were eluted in a linear gradient with acetonitrile (ACN) containing 0.1% ammonium hydroxide at a flow rate of 0.3 ml/min for 5 min. The metabolites were analyzed by Q-TOF MS (Waters) in negative electrospray ionization (ESI) mode. The capillary and sampling cone voltages were set at 0.5 kV and 16 V, respectively. The desolvation flow was 800 L/h at a temperature of 450 °C, and the source temperature was 100 °C. The MS data were collected in the m/z 50−1200 range with a scan time of 0.5 seconds. The LC-MS data were analyzed by MassLynx (Version 4.1, Waters, USA). The relative intensity of interested metabolites was compared by TargetLynx, with a mass window of 0.05 Dalton.

Measurements of lactate concentration

Virus-infected sphere cells were seeded on a 12-well plate with defined serum-free selection medium at an initial concentration of 1×10^6^ cells per well. The supernatants were collected after 24 hr and series diluted with medium in 96-well plates. The lactate concentration was then assayed using the lactate assay kit (Eton Bioscience), according to manufacturer’s instructions. Relative lactate levels were normalized to the amount of protein.

Proteomics analysis of Plasma Membrane protein

SAS sphere cells were isolated, digested, fracture, and analyzed with liquid chromatography coupled with tandem mass spectrometry (LC-MS/MS) as previously described.^5^

Flow cytometric analysis

Single-cell suspensions were prepared and 1×10^6^ cells incubated with antibodies in PBS containing 2% FBS for 30 min at 4°C. The antibodies used in this study included CD44 (Catalog No.FAB5088; R&D), Grp78 (BD Transduction Laboratories™), Glut3 (Catalog No.BAM1415; R&D) and Ck18 (Catalog No.MAB3234; Millipore) to determine the adhesion molecule expression on the cell surface. The purified mouse IgG isotype was used as a control. Primary antibodies were revealed with APC-conjugated goat anti-mouse IgG (BD Biosciences).

The Annexin V Staining kit (Catalog No.88-8005; eBioscience) was used to examine apoptosis. The protocol was according to the instructions included in the Annexin V Staining kit. For cell death, the cells were collected and incubated with 1 μg/ml propidium iodide (PI) at room temperature for 30 mins and analyzed by flow cytometry. All analyses were performed using a FACScanto flow cytometry System (Becton Dickinson). The antibodies used for the flow cytometric analysis are listed in Table S3.

Xenograft Mouse Model

All animal studies were approved and conducted under the guidelines by National Yang-Ming University’s Institutional Animal Care and Use Committee (IACUC) (approval No. 1031278). Virus-infected sphere cells were trypsinized to a single cell suspension and resuspended in 100 μL medium containing Matrigel ((1:1, v/v; BD Bioscience, San Diego, CA, USA) and subcutaneously injected into the back of nude mice (6–8 weeks). Tumor volume was calculated using the following formula: (Length × Width 2) / 2.

Analyses of Nrf2 and its target genes in human tumors and cell lines by bioinformatics and microarray approaches

Gene expression profiling was analyzed using Affymetrix Human Genome U133plus2.0 for SAS HNSCC cells (GEO datasets, GSE35603). The Cancer Genome Atlas (TCGA) dataset (http://tcga-data.nci.nih.gov/tcga/) was used to mining gene expression with clinical annotations. The SurvExpress and the Cancer Proteome Atlas (TCPA) website database are a large collection of published microarray and proteome datasets, respectively, for assessing the relationship between gene expression and prognostic value.^6, 7^ The accession numbers of our subjects obtained were GSE10300, GSE26549, E-MTAB-1328.

Chromatin Immunoprecipitation (ChIP) sequencing and data analysis

ChIP-Seq was used to analyze the binding patterns of Nrf2 in SAS or SAS-S cells. ChIP assays were performed using EZ-ChIP^TM^ (Cat#17-371; Millipore, Billerica, MA) according to the manufacturer's instructions. SAS or SAS-S cells were fixed in 1% formaldehyde in cell growth media for 10 min. Cells were then sonicated to shear DNA. Upon sonication, a part of the supernatants was then processed to as Input samples. Then the other supernatants were immunoprecipitated with either control rabbit IgG, or polyclonal antibody Nrf2. Protein A/G plus agarose beads were then added to each reaction mixture. Thereafter, protein/DNA complexes were eluted and checked for 100 to 300-bp fragment enrichments using 2% agarose gel electrophoresis (data not shown).

For ChIP-seq, the sequences of DNA fragments were determined using Genome Analyzer GAIIx (Illumina, Inc., San Diego, CA) at a read length of 100 bp. The sequencing reads were mapped to the GRCh38 genome assembly. The data were further analyzed using the Partek genomics suite (Partek Inc., St. Louis, MO). The accession number of our subject was GSE95818.

Ingenuity Pathway Analysis

The statistically significant changes in gene expression were used to determined signatures for network coding that were identified using the Ingenuity Pathway Analysis (IPA) analyses. The Ingenuity Pathways Knowledge Base (<http://www.ingenuity.com/>) is a global database of knowledge on biological networks, with annotations by experts.^8^ Enrichment significance is expressed as the negative Log10 of the p-value, which is represented on the y-axis. The p-value was calculated using the right-tailed Fisher’s Exact Test. The ratio was calculated as the number of predicted targets /total gene number of each pathway.

Gene Set Enrichment Analysis (GSEA)

Pathway enrichment analysis was performed by using the GSEA program (<http://software.broadinstitute.org/gsea/downloads.jsp>). The differentially expressed genes were then subjected to GSEA program. The gene sets from MSigDB database (<http://software.broadinstitute.org/gsea/msigdb>) were used for GSEA program. A normalized enrichment score (NES) was the statistic for examining the number of the genes present in the gene set. The Benjamini–Hochberg procedure was used to compute the false discovery rate (FDR) from the P values.^9^

**References**

1. Okumura K, Konishi A, Tanaka M, Kanazawa M, Kogawa K, Niitsu Y. Establishment of high- and low-invasion clones derived for a human tongue squamous-cell carcinoma cell line SAS. *J Cancer Res Clin Oncol* 1996, **122**(4)**:** 243-248.

2. Chiou SH, Yu CC, Huang CY, Lin SC, Liu CJ, Tsai TH*, et al.* Positive correlations of Oct-4 and Nanog in oral cancer stem-like cells and high-grade oral squamous cell carcinoma. *Clinical cancer research : an official journal of the American Association for Cancer Research* 2008, **14**(13)**:** 4085-4095.

3. Wu MJ, Jan CI, Tsay YG, Yu YH, Huang CY, Lin SC*, et al.* Elimination of head and neck cancer initiating cells through targeting glucose regulated protein78 signaling. *Molecular cancer* 2010, **9:** 283.

4. Chang CW, Chen YS, Chou SH, Han CL, Chen YJ, Yang CC*, et al.* Distinct subpopulations of head and neck cancer cells with different levels of intracellular reactive oxygen species exhibit diverse stemness, proliferation, and chemosensitivity. *Cancer research* 2014, **74**(21)**:** 6291-6305.

5. Han CL, Chien CW, Chen WC, Chen YR, Wu CP, Li H*, et al.* A multiplexed quantitative strategy for membrane proteomics: opportunities for mining therapeutic targets for autosomal dominant polycystic kidney disease. *Molecular & cellular proteomics : MCP* 2008, **7**(10)**:** 1983-1997.

6. Aguirre-Gamboa R, Gomez-Rueda H, Martinez-Ledesma E, Martinez-Torteya A, Chacolla-Huaringa R, Rodriguez-Barrientos A*, et al.* SurvExpress: an online biomarker validation tool and database for cancer gene expression data using survival analysis. *PLoS One* 2013, **8**(9)**:** e74250.

7. Li J, Lu Y, Akbani R, Ju Z, Roebuck PL, Liu W*, et al.* TCPA: a resource for cancer functional proteomics data. *Nat Methods* 2013, **10**(11)**:** 1046-1047.

8. Pinatel EM, Orso F, Penna E, Cimino D, Elia AR, Circosta P*, et al.* miR-223 is a coordinator of breast cancer progression as revealed by bioinformatics predictions. *PloS one* 2014, **9**(1)**:** e84859.

9. Subramanian A, Tamayo P, Mootha VK, Mukherjee S, Ebert BL, Gillette MA*, et al.* Gene set enrichment analysis: a knowledge-based approach for interpreting genome-wide expression profiles. *Proceedings of the National Academy of Sciences of the United States of America* 2005, **102**(43)**:** 15545-15550.
