# Supplementary material for: ROS-independent ER stress-mediated NRF2 activation promotes warburg effect to maintain stemness-associated properties of cancer-initiating cells
Source: Cell Death Dis. 2018 Feb 7;9(2):194. doi: 10.1038/s41419-017-0250-x (PMC5833380; doi:10.1038/s41419-017-0250-x)
Supplement: Supplementary file 3 — Supplemental table [file 41419_2017_250_MOESM3_ESM.docx]

**Supplemental table**

**Table 1. shRNA used in the present study**

| shRNA | Clone ID | Target sequence | Vector |
| --- | --- | --- | --- |
| shHK2-1 | TRCN0000232925 | GGGACTTTGATATCGACATTG | pLKO_TRC005 |
| shHK2-2 | TRCN0000232927 | TGACGACAGCATCATTGTTAA | pLKO_TRC005 |
| shPFKFB3-1 | TRCN0000314746 | CGTGTCGGTTCCATTCCATTT | pLKO_TRC005 |
| shPFKFB3-2 | TRCN0000314747 | CGTGACTGTTTGGTGCATCTT | pLKO_TRC005 |
| shNrf2-1 | TRCN0000284999 | CCGGCATTTCACTAAACACAA | pLKO_TRC005 |
| shNrf2-2 | TRCN0000273494 | AGTTTGGGAGGAGCTATTATC | pLKO_TRC005 |
| shGrp78-1 | TRCN0000218611 | AGATTCAGCAACTGGTTAAAG | pLKO_TRC005 |
| shGrp78-2 | TRCN0000257180 | GGAACCATCCCGTGGCATAAA | pLKO_TRC005 |
| shPERK-1 | TRCN0000262373 | TGCATCTGCCTGGTTACTTAA | pLKO_TRC005 |
| shPERK-2 | TRCN0000262379 | TAGCAGCAATCCCTAATATAT | pLKO_TRC005 |

**Table 2. Primers used in the present study**

| **Gene** | **Direction** | **Sequences (5’ - 3’)** |
| --- | --- | --- |
| Slc2a1 | Forward | CAGTTCGGCTATAACACTGGTG |
|  | Reverse | GCCCCCGACAGAGAAGATG |
| LDHA | Forward | CATTGTCAAGTACAGTCCACACT |
|  | Reverse | TTCCAATTACTCGGTTTTTGGGA |
| MCT4 | Forward | TCACGGGTTTCTCCTACGC |
|  | Reverse | GCCAAAGCGGTTCACACAC |
| PFKFB3 | Forward | CCCAGAGCCGGGTACAGAA |
|  | Reverse | GGGGAGTTGGTCAGCTTCG |
| HK2 | Forward | GAGGGCAGATCTCTGAGACG |
|  | Reverse | GCACACTGTCTTGACGAGGA |
| PFK1 | Forward | GGCAC CCTCT CCATT TGATA |
|  | Reverse | GCACA CAAAT GGAAT CATCG |
| PGK1 | Forward | GCTCT CATGG ATGAG GTGGT |
|  | Reverse | CTTCC AGGAG CTCCA AACTG |
| ENOS | Forward | CTCCG TGACC GAGTC TCTTC |
|  | Reverse | CCAGT CTTGA TCTGC CCAGT |

**Table 3. Information for antibodies used in the experiments**

| Antibody | Host | Dilution | Supplier | Cat. No. |
| --- | --- | --- | --- | --- |
| Nrf2 C-20 | rabbit | 1:1000 | Santa Cruz Biotech | sc-722 |
| p-Nrf2 | rabbit | 1:5000 | Abcam | ab76026 |
| p-Nrf2* | rabbit | 1:100 | Abcam | ab76026 |
| Nrf2^#^ | rabbit | 2 μg | Santa Cruz Biotech | sc-722x |
| Actin | mouse | 1:5000 | Millipore | MAB1501 |
| Oct-3/4 | rabbit | 1:500 | Santa Cruz Biotech | sc-9081 |
| Nanog | rabbit | 1:1000 | Millipore | AB9220 |
| HK2 | goat | 1:1000 | Santa Cruz Biotech | sc-6521 |
| PFKFB3 | rabbit | 1: 1000 | Proteintech | 13763-1-AP |
| LDHA | rabbit | 1:10000 | Cell Signaling | 3582 |
| PGK1 | rabbit | 1:1000 | Santa Cruz Biotech | sc-17943 |
| PDK1 | rabbit | 1:20000 | Santa Cruz Biotech | sc-7140 |
| IgG^#^ | rabbit | 2 μg | Santa Cruz Biotech | sc-2027 |
| Grp78 | mouse | 1:5000 | [BD Biosciences](http://www.bdbiosciences.com/ptProduct.jsp?prodId=25684) | 610978 |
| PERK | rabbit | 1:1000 | Cell Signaling | 3192 |
| p-PERK | rabbit | 1:1000 | Santa Cruz Biotech | sc-32577 |
| p-PERK^#^ | rabbit | 1:100 | Santa Cruz Biotech | sc-32577 |

* IHC; ^#^ChIP; others, Western blot analysis
